# Atomic Force Microscopy (AFM)-Based Metrology for Advanced Etching in Three-Dimensional Integrated Circuits

**DOI:** 10.3390/mi17050565

**Published:** 2026-05-01

**Authors:** Jing Chang, Shixuan Wang, Shizhen Liang, Xihao Feng, Wei Zhao

**Affiliations:** 1 College of Chemistry and Chemical Engineering, Xi’an University of Science and Technology, Xi’an 710054, China; jchang@xust.edu.cn (J.C.); 22415030123@stu.xust.edu.cn (S.L.); 18591868600@163.com (X.F.); 2 China Development Strategy Institute for Building Materials Industry, Beijing 100035, China; wangshixuan2022@126.com

**Keywords:** atomic force microscopy (AFM), three-dimensional integrated circuits (3D ICs), etching technologies, semiconductor metrology

## Abstract

Fueled by the push for “More than Moore”, three-dimensional integrated circuits (3D ICs) have become a backbone of next-generation electronics. Their complex architectures place unprecedented demands on etching technologies, which must now deliver atomic precision, stringent high-aspect-ratio (HAR) control, and virtually damage-free profiles. Meeting these challenges requires metrology capable of true 3D, quantitative analysis at the nanoscale. Atomic force microscopy (AFM) has proven essential in this regard, offering non-destructive, sub-nanometer characterization that other techniques cannot provide. This review systematically examines AFM’s pivotal role in advancing key etching processes for 3D ICs, including deep reactive ion etching of through-silicon vias (TSVs), atomic layer etching (ALE), and cryogenic plasma etching. We detail AFM’s unique contributions to quantifying sidewall roughness, verifying etch-per-cycle rates, and assessing surface damage. We also discuss how recent innovations, such as tilting-AFM, HAR probes, and automated inline systems, are overcoming traditional barriers in throughput and access to sidewalls and deep trenches. Looking forward, the integration of AFM with optical metrology, machine learning, and multi-scale modeling opens a path toward truly autonomous process control and optimization. As such, AFM stands as an indispensable tool for developing and refining the etching processes that underpin next-generation 3D semiconductor manufacturing.

## 1. Introduction

The relentless pursuit of higher performance, lower power consumption, and greater functional density has driven a paradigm shift in microelectronics—from planar integrated circuits (ICs) to three-dimensional (3D) integration. This transition is not merely an incremental advancement but a necessary response to the diminishing returns of conventional Moore’s Law scaling. At the heart of this shift, advanced etching technologies have evolved from mere pattern transfer tools into precision sculptors of complex, HAR micro/nanostructures that define 3D IC architectures.

For over four decades, planar Metal-Oxide-Semiconductor Field-Effect Transistor (MOSFET) scaling powered semiconductor progress, but it now faces insurmountable physical and economic barriers [1]. Physically, as process nodes approach and exceed the 1-nm regime, geometric shrinkage of planar structures becomes unviable due to severe short-channel effects (SCEs), which weakens gate control over the channel and led to increased off-state leakage [2,3]. The planar Complementary Metal-Oxide-Semiconductor (CMOS) gate length limit has effectively stalled at the ~22 nm node, where leakage power already accounts for 20–30% of total consumption [1,2]. Scaling the gate dielectric thickness (e.g., SiO_2_) to mitigate SCEs has also reached a practical limit of ~1.2 nm [1,2], while parasitic resistance worsens and incremental switching speed improvements no longer justify the complexity of innovations like strained Si or high-κ dielectrics [2]. Economically, planar scaling has become prohibitive: a 3-nm production line costs over $20 billion, with 2-nm investments projected to rise by 50% [4]. Falling transistor costs no longer offset soaring R&D and manufacturing expenses, compounding diminishing returns [2].

In response, 3D ICs, chiplets, and system-in-package (SiP) solutions have emerged as alternatives to conventional system-on-chip (SoC) designs [3]. 3D integration enables heterogeneous stacking of disparate technology nodes, reducing interconnect latency and power consumption while boosting functional density far beyond planar IC limits [5,6]. Key 3D integration paradigms include: 2.5D interposers, 3D stacked ICs (3D-SICs) with through-silicon vias (TSVs), monolithic 3D integration (M3D), and Face-to-Face (F2F) 3D ICs. TSVs are indispensable for 3D-SICs and advanced packaging, enabling vertical stacking to support high-bandwidth memory (HBM), AI accelerators, and 6G communication chips [7].

Etching has become a foundational enabler for 3D features, evolving from pattern transfer to the sculpting of complex HAR micro/nanostructures. As 3D IC complexity grows, etching processes must meet the following four tightly coupled demands central to cutting-edge semiconductor development.

(1) Precise Profile Control. Achieving vertical sidewalls, regulating tapering, and minimizing scalloping are critical for fabricating high-aspect-ratio (HAR) structures via deep reactive ion etching (DRIE), especially the Bosch process [8]. Innovations in process integration (e.g., hybrid lithography [EBL + UVL] combined with Inductively Coupled Plasma (ICP)-DRIE) enable effective sidewall taper control [8,9]. To address inherent Bosch process scalloping, a multistep plasma etching strategy forms regular semicircular gaps on HAR microhole sidewalls, minimizing irregular scalloping and supporting subsequent corner lithography [9]. At the atomic scale, cyclic atomic-level etching (ALE) meets the ultra-high precision profile control requirements of next-generation 3D ICs, achieving atomic-level vertical sidewall regulation while minimizing lattice damage and surface roughening [10]. This stringent requirement for profile fidelity translates directly into a critical metrology challenge: quantifying sidewall angle (SWA), scallop amplitude/periodicity, and line edge roughness (LER) on near-vertical, deep trench sidewalls with sub-nanometer precision.
(2) High Selectivity. Etching the target material while minimizing erosion of masking layers and underlying substrates is a fundamental prerequisite for fabricating complex 3D IC architectures [11,12,13]. Pulsed plasma etching enhances selectivity by regulating the dynamic balance between chemical adsorption and ion bombardment [11]. Innovative paradigms further optimize selectivity: phase-controlled etching in triode-type capacitively coupled plasmas (CCP) modulates plasma density and sheath thickness [6], with Δphase = 90° improving PR neck critical dimension (CD) by 14% and SiO_2_/Si_3_N_4_ mold bottom CD by 32% [14]; second-generation cryo-etch (HF plasma) for 3D NAND ONON stacks doubles etch rate vs. first-generation NF_3_/H_2_ plasma, with pure HF boosting Si_3_N_4_ selectivity [15]. Process parameters directly govern selectivity [12,13], achieved through synergistic optimization of plasma chemistry, masking materials, and process parameters [15]. Achieving high selectivity demands metrology capable of measuring etch depths on different material regions with sub-nanometer vertical resolution, thereby allowing etch rates to be distinguished. Furthermore, verifying that the mask layer retains its critical dimensions and integrity after aggressive etching requires high-resolution, non-destructive profiling of both the etched feature and the remaining mask.
(3) Low Damage. Minimizing lattice damage, surface roughening, and charge-induced damage are indispensable for 3D ICs with HAR structures (e.g., TSVs, monolithic integration), as such damages degrade carrier mobility, increase leakage current, and reduce reliability. Lattice damage (caused by ion bombardment in conventional plasma etching) is mitigated by emerging paradigms like ALE and neutral beam etching (NBE) [16,17,18,19]. Precisely quantifying key damage indicators, such as surface roughening, is essential for guiding the optimization of low-damage etching processes. Accurate measurement of these parameters provides critical insights to drive process refinement and help mitigate lattice or charge-induced damage.
(4) Backend-of-line (BEOL) Compatibility. Ensuring compatibility with temperature-sensitive BEOL materials and structures is a pivotal constraint for advancing 3D ICs, especially as logic nodes scale to 7 nm and beyond [19]. Primary BEOL temperature-sensitive materials include porous SiOCH dielectrics and SiCOH-based ultra-low-κ (ULK) films, which are widely adopted in advanced interconnect nodes but highly sensitive to conventional fluorocarbon (FC) etch chemistry [19,20], causing dielectric constant increase, reliability degradation and irreversible structure damage [20]. Conventional continuous-wave (CW) plasma etching exacerbates RIE lag and severe ULK damage [19,20,21]. Emerging solutions include quasi-atomic layer etching (QALE), which separates radical absorption from ion bombardment, suppressing RIE lag to ~1 nm, minimizing ULK damage and improving hard mask selectivity. For BEOL compatibility, metrology needs to non-destructively evaluate plasma-induced damage in porous low-κ dielectrics (e.g., changes in roughness, modulus, carbon depletion) while also confirming that RIE lag is held to ~1 nm, a task that demands sub-nanometer vertical resolution on structures with different materials.

**Figure 1:**
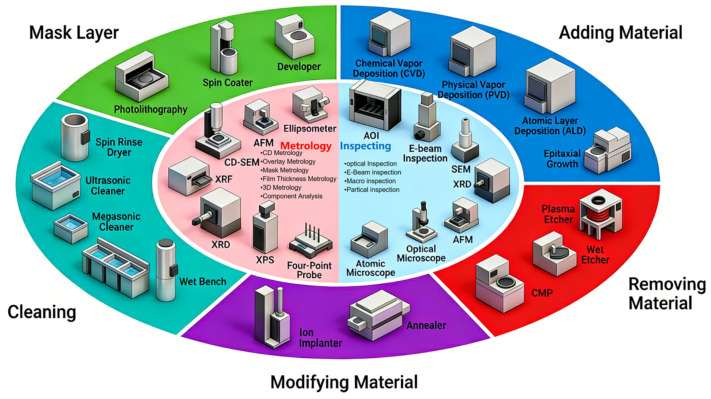
Integration of surface metrology tools in a semiconductor production line for acquiring precise physical dimensions and material properties (e.g., film thickness, critical dimension, etch depth, surface topography, chemical composition).

Meeting these four tightly coupled demands is essential, yet it also highlights a fundamental challenge: how do we verify such nanoscale features? This leads to the central metrology gap: the need for true 3D observation at the nanoscale. Advanced manufacturing, including 3D chip/wafer stacking and advanced etching processes for 3D ICs, places stringent demands on measurement technologies, requiring ultra-high precision, non-contact operation, high throughput, and real-time adaptability [22,23]. As illustrated in Figure 1, a modern semiconductor production line integrates numerous metrology and inspection tools alongside manufacturing processes to monitor critical parameters in real time. However, traditional metrology methods—predominantly consisting of contact-based coordinate measurement and standalone offline systems—possess inherent limitations that render them unable to satisfy these critical requirements [24,25]. Conventional contact-based techniques cause mechanical wear or surface damage to delicate micro-nano structures and heavily rely on skilled operators, requiring manual intervention (e.g., removing workpieces from the production line) that prolongs inspection cycles, disrupts process monitoring, and compromised efficiency [22,24]. As standalone systems, traditional metrology is confined to post-process part inspection, failing to encompass manufacturing processes and equipment—restricting real-time process control and efficient statistical analysis, leaving dynamic quality variations unaddressed.

For specific scenarios such as TSV etching in 3D ICs, traditional destructive techniques (e.g., cross-sectional SEM) are only viable for process development and cannot support in-line or non-destructive measurement; conventional optical microscopy, meanwhile, is limited by the opaque nature of silicon wafers, making it incapable of characterizing HAR features [23]. Even non-contact optical tools like early spectral reflectometers or interferometers, without targeted optimization, struggle to achieve one-shot measurement of critical TSV parameters (e.g., via depth, bottom roughness, bottom profile) or adapt to cross-scale measurement needs—from macro-structural alignment to micro-nano surface characterization [23].

Collectively, these limitations create a significant metrology gap: traditional characterization methods become bottlenecks in the development of advanced manufacturing processes due to their shortcomings in non-destructive capability, throughput, statistical relevance, and adaptability to complex 3D structures [22].

To bridge this critical gap, advanced metrology solutions capable of nondestructive, high-precision 3D characterization at the nanoscale are urgently needed—requirements that position atomic force microscopy (AFM) as a compelling candidate.

## 2. Fundamentals of AFM for 3D Semiconductor Metrology

Against the backdrop of 10 nm-node semiconductor scaling and 3D stacked architectures (e.g., 3D memories, Fin Field-Effect Transistor (FinFETs)), nanoscale 3D metrology demands high-precision, non-destructive 3D profiling. AFM has emerged as an indispensable core tool for this task, with inherent advantages of high accuracy, 3D imaging capability, sub-nm spatial resolution, and non-destructivity, which perfectly addresses the metrological challenges of device miniaturization and 3D stacking [26]. Although conventional AFM has long faced a throughput bottleneck that limited its application in high-volume manufacturing, recent innovations in high-speed scanning, parallel probe architectures, automated inline systems, and machine-learning-assisted imaging have significantly alleviated this limitation, making AFM increasingly viable for high-volume manufacturing.

This review systematically explores AFM’s role as a 3D metrology enabler for 3D IC advanced etching processes, covering fundamental principles, core technologies, critical applications, and unresolved challenges.

### 2.1. Principles of Operation

Invented by Binnig et al. in the 1980s [27], AFM has become one of the most widely used scanning probe techniques, which can image both conducting and insulating surfaces with atomic resolution—a critical advantage for semiconductor metrology where diverse material stacks are involved.

**Figure 2:**
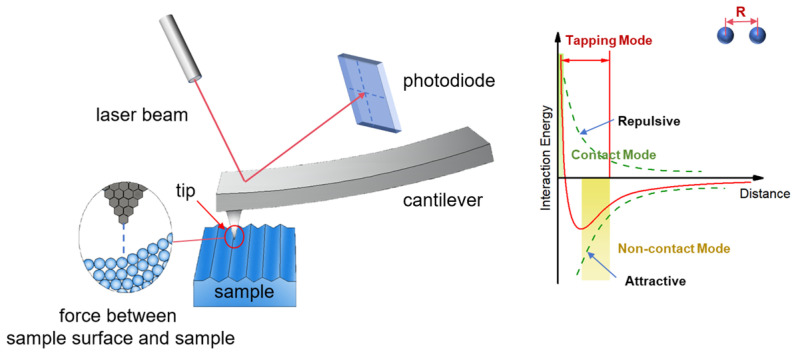
Schematic of an atomic force microscope (AFM).

In AFM (Figure 2), a sharp tip on a cantilever scans across the sample surface. The tip–sample interaction force is measured via cantilever deflection, and a feedback loop maintains a constant force during scanning by adjusting the height of the translation stage, thereby generating a topographic image. Image contrast arises because the tip–sample force depends on both material properties and separation distance. To date, in most applications, image contrast is obtained from the very short-range Born repulsion that occurs when the electron orbitals of tip and sample overlap. Beyond topography, AFM can perform “force measurements” to probe mechanical, chemical, or electrical interactions at the nanoscale [28]. This capability is particularly valuable for 3D IC etching metrology, where features such as HAR trenches, FinFET gate structures, and TSV sidewalls exhibit not only complex topography but also process-induced variations in material properties (e.g., surface damage, residue contamination, or modulus changes in low-k dielectrics). Characterizing these features therefore requires more than geometric profiling, which demands the nanoscale interaction sensitivity that AFM force measurements uniquely provide.

For semiconductor etching metrology, AFM offers four primary AFM operation modes, each with distinct trade-offs for measuring etched features in 3D IC structures:

- Contact Mode: In this mode, the AFM tip remains in direct physical contact with the sample surface during scanning. It offers high resolution and fast imaging but suffers from sample damage and tip wear. For etched semiconductor features, this is particularly problematic when measuring HAR structures where tip geometry changes can significantly impact measurement accuracy [29].
- Tapping Mode: The tip oscillates or “taps” the surface, which reduces damage compared to contact mode and can provide high resolution. For semiconductor metrology, tapping mode offers a balance between resolution and tip preservation, making it suitable for repeated measurements on production wafers where tip lifetime is a critical economic factor [30].
- Non-Contact Mode: The tip oscillates above the sample surface without establishing direct physical contact, sensing long-range attractive forces (e.g., van der Waals forces) to maintain a constant tip–sample separation of several nanometers. This eliminates tip wear and sample damage entirely, preserving tip sharpness for extended periods. For semiconductor etching metrology, non-contact mode enables non-destructive measurement of sensitive materials (e.g., EUV photoresist trenches, TSV structures) with sub-angstrom noise levels [31,32]. While offering lower resolution than contact or tapping modes, non-contact mode is particularly valuable for process control applications where measurement consistency across multiple wafers is essential [33].
- PeakForce Tapping Mode: The probe oscillates at a frequency well below its resonance, and the system measures and controls the instantaneous peak force between the tip and sample during each contact cycle. Through precise force feedback, it enables high-resolution imaging at extremely low contact forces (down to the piconewton range), significantly reducing tip wear and damage to delicate samples [34]. For semiconductor etching metrology, PeakForce Tapping not only enables nondestructive characterization of HAR structures (e.g., deep trenches, TSVs) and sensitive materials (e.g., EUV photoresists, low-κ dielectrics) but also captures force-distance curves at each pixel, providing quantitative nanomechanical properties such as modulus, adhesion, deformation, and dissipation [33]. This multidimensional information is critical for assessing process-induced mechanical changes (e.g., low-κ dielectric embrittlement) and identifying defects, making it an ideal tool for both process development and in-line monitoring.

These four basic modes constitute the core imaging and measurement capabilities of AFM for semiconductor applications, providing the topographical foundation essential for characterizing etched structures. However, the increasing complexity of 3D ICs demands more than geometric profiling—a need that has driven the development of advanced AFM-based techniques.

### 2.2. Multifaceted Strengths of AFM: Beyond Topography to Material Property Mapping

Building on these fundamental imaging modes, advanced AFM techniques have been developed to extend measurement capabilities beyond topography to quantitative mechanical, electrical, and chemical characterization, addressing the multifaceted demands of modern semiconductor etching metrology where nondestructive imaging alone is insufficient. Among these, scanning spreading resistance microscopy (SSRM), scanning capacitance microscopy (SCM), and photo-induced force microscopy (PiFM) provide complementary capabilities—respectively offering carrier concentration profiling, pn junction mapping, and nanoscale chemical identification—capabilities that are essential for etching metrology, as elaborated below.

- Scanning Spreading Resistance Microscopy: SSRM is a contact-mode-based AFM technique for high-resolution quantitative mapping of two-dimensional carrier distributions in semiconductor devices [35,36]. Its operating principle involves scanning a conductive probe under high force (typically generating 8–12 GPa of pressure), which induces a phase transformation in the silicon beneath the tip to metallic β-Sn, forming a minute virtual electrode. By applying a DC bias between the probe and sample, the spreading current (or resistance) through this metallized region is measured, which directly correlates with local carrier concentration. For semiconductor etching metrology, SSRM provides quantitative carrier concentration profiles with nanometer spatial resolution, enabling verification of doping integrity in source/drain regions after etching, analysis of doping uniformity in 3D structures such as FinFETs or gate-all-around (GAA) devices, and diagnosis of doping anomalies or failures induced by etching processes.
- Scanning Capacitance Microscopy: SCM is a variant of contact-mode AFM used to image two-dimensional carrier distributions in semiconductor devices [37]. The technique is based on measuring the differential capacitance (dC/dV) of the metal-oxide-semiconductor (MOS) structure formed by the metallized probe, the thin native oxide on the sample surface, and the semiconductor substrate. When an AC bias is applied, carrier accumulation and depletion in the semiconductor modulate the total capacitance; the magnitude of this modulation correlates with local carrier concentration, while its phase indicates carrier type (p-type or n-type). For semiconductor etching metrology, SCM offers high-resolution identification of pn junction locations and contours, enabling two-dimensional mapping of doped regions. This capability is valuable for analyzing doping integrity in well regions and lightly doped drain (LDD) structures after etching, as well as for localizing ion implantation-related defects.
- Photo-Induced Force Microscopy: PiFM is an advanced technique that combines the high spatial resolution of non-contact AFM with the chemical specificity of infrared spectroscopy [38]. Its operating principle involves focusing a tunable infrared laser at the AFM tip apex, generating a tip-enhanced optical field that interacts with the sample to produce a photo-induced force detected by the AFM probe. By scanning the infrared laser wavelength, nanoscale spectra directly correlating with Fourier-transform infrared spectroscopy (FTIR) are obtained, enabling precise chemical identification. For semiconductor etching metrology, PiFM simultaneously acquires topographical and chemical information under nondestructive, non-contact conditions with spatial resolution better than 5 nm. This makes it a powerful tool for analyzing organic residues in EUV lithography processes, identifying the chemical composition of sub-20 nm defects, evaluating cleaning process effectiveness, and characterizing monolayer films—addressing the limitations of conventional SEM/EDS methods, which lack molecular information and risk electron beam damage [39].

### 2.3. Specialized AFM Techniques for Advanced Integrated Circuit Metrology

The escalating complexity of 3D IC architectures (e.g., FinFETs at the 1× node and beyond) demands precise 3D metrology for critical features like Gate-Fin corner poly-footing and vertical sidewall LER. To address these metrology challenges and enable true three-dimensional characterization, several specialized AFM techniques have been developed, each offering unique capabilities for nondestructive, SI-traceable 3D characterization with sub-nm resolution, low noise, and high repeatability across diverse 3D IC structures [40].

**Figure 3:**
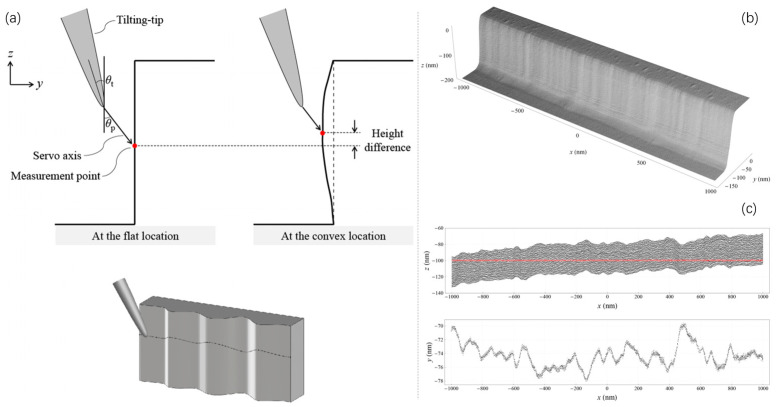
(**a**) Schematic of line edge roughness measurement on a vertical sidewall using a tilting-mAFM; (**b**) AFM image of a Si line pattern from one side; (**c**) Height–constant sidewall profile formed by points picked up from multiple fast-scan profiles of the AFM data, and the height constant sidewall profile of the red strip projected onto xy plane. Reproduced from ref. [42] with permission.

- Metrological tilting-AFM (tilting-mAFM): As a key breakthrough, tilting-mAFM overcomes conventional AFM’s finite probe cone angle (20–30°) via a 16-max tip-tilting mechanism, enabling vertical sidewall topography measurement [32]. Equipped with SI-traceable laser interferometry, it achieves sub-nm repeatability (x = 0.12 nm, y = 0.20 nm, z = 0.20 nm for sidewalls) and 0.09° SWA repeatability; its inclining servo control and backward probe lifting ensure stable, damage-free scanning [41]. For LER metrology, tilting-mAFM delivers <0.5 nm sidewall resolution, with high-frequency power spectral density (PSD) noise orders of magnitude lower than CD-SEM [42].
- Three-dimensional atomic force microscopy (3D-AFM): While tilting-AFM enables sidewall access, 3D-AFM provides comprehensive profile information for microstructures, directly measuring parameters such as SWA, linewidth variability, sidewall roughness (SWR), and CDs—including undercuts and steep sidewalls that 2D techniques cannot capture [26,42]. Some 3D-AFM systems operate in contact mode, with non-contact mode under development. For 3D IC etching applications, 3D-AFM delivers the full-profile information essential for process optimization. As an example of sidewall-sensitive measurement, Figure 3 illustrates how tilting-mAFM captures vertical sidewall topography and line edge roughness, complementing the full-profile capability of 3D-AFM.
- High-speed metrology large-range AFM (HS Met. LR-AFM): Beyond precision, AFM also breaks speed and range limits. Ongoing efforts focus on improving throughput, precision (targeting 0.2 nm^2^ for beyond-1x nodes), and adapting to ultra-HAR structures [43]. HS Met. LR-AFM reaches a scan rate of 1 mm/s (20× faster than conventional AFM) with ±0.7 nm step repeatability, significantly reducing drift and calibration costs [44]. 3D-printed AFM probes further expand AFM’s utility by enabling ultra-high-speed imaging (≈10× higher bandwidth than silicon probes) and programmable vibrational modes. Ultra-large scan size AFM (ULSS-AFM) achieves 1 × 1 mm^2^ stitchless scanning (two orders of magnitude larger than conventional AFM), enabling cross-scale metrology from nanoscale TSVs to mm-scale chip flatness [44,45].
- Further advancements in accuracy: AFM’s value in semiconductor metrology has been enhanced by bottom-up traceable tip calibration and convolution correction models that eliminate tip-induced distortion [40,43]. Accuracy is ensured through rigorous tip geometry characterization—using edge reversal methods for tip radius evaluation and reference artifact scanning for included angle measurement—combined with traceability to silicon lattice constants. These approaches address the inherent convolution of tip and sample profiles [40,43].

In summary, these specialized AFM techniques deliver true 3D, nondestructive characterization—covering vertical sidewalls, full profiles, high-speed large-area scanning, and traceable accuracy—thereby addressing critical metrology gaps in advanced 3D IC manufacturing.

### 2.4. Comparison of AFM with Other Semiconductor Metrology Techniques

To contextualize AFM’s role in 3D IC etching metrology, it is helpful to assess its characteristics with other mainstream techniques used in semiconductor device fabrication, including optical techniques, scanning electron microscopy (SEM), and transmission electron microscopy (TEM). Each technique is built upon distinct physical principles and exhibits complementary strengths and limitations across key parameters such as resolution, throughput, sample preparation, and 3D profiling capability, as summarized in Table 1 [22,26,46].

Optical metrology encompasses a family of non-contact techniques widely used in semiconductor manufacturing, ranging from traditional optical microscopy for rapid visualization to scatterometry, interferometry, and confocal microscopy for high-precision dimensional and topographic measurements [22,26,46]. These methods enable high-speed, in-line determination of critical geometric parameters such as linewidth, SWA, and film thickness, as well as surface topography, but often rely on indirect model-based inference [46]. However, most optical techniques face inherent limitations, including diffraction-limited resolution in imaging-based methods and a reliance on indirect inverse modeling [26,47,48]. Scatterometry, a widely adopted non-imaging technique, exemplifies both the strengths and the challenges of optical metrology: it extracts ensemble-averaged dimensional information from periodic arrays by analyzing changes in the polarization and intensity of scattered light, offering advantages in speed and non-destructiveness [46]. Yet it shares the broader challenges of optical metrology: relying on indirect inverse modeling and parametric libraries, solutions can be non-unique, and parameter correlations increase measurement uncertainty [46]. Furthermore, they are sensitive to surface reflectivity, ambient conditions, and are limited by line-of-sight, making it difficult to accurately characterize complex 3D structures such as gate-all-around nanowires or through-silicon vias [22,24,46]. In contrast, AFM provides a direct, model-independent measurement of local topography. This distinction establishes AFM as a critical reference metrology tool for reducing uncertainty in hybrid metrology workflows. Studies show that incorporating CD-AFM data (for SWA and LER) into scatterometry models can reduce CD measurement uncertainty by as much as 4 nm [46].

SEM is a versatile tool for dimensional metrology in semiconductor manufacturing. However, conventional SEM systems are not ideally suited for in-line production environments: they typically operate at higher beam energies that can cause sample damage and charging, require frequent calibration and manual intervention, and lack the high throughput and measurement repeatability demanded by high-volume manufacturing. To address these limitations, specialized CD-SEM systems have been developed for in-line CD measurements, offering high throughput and seamless integration with fabrication infrastructure [46,49,50]. CD-SEM provides top-down images with sub-nanometer lateral resolution and, when coupled with model-based libraries or tilt-beam capability, can extract three-dimensional information such as SWA, with a demonstrated precision of approximately 0.1–0.2° when averaged over multiple features [46,51]. However, as a fundamentally 2D projection technique, its ability to characterize sub-5 nm features in FinFET and nanowire devices relies on coupling measurements with simulation and modeling to optimize measurement conditions and results interpretation [49,52]. Moreover, measurement accuracy can be affected by error sources such as drift, vibration, charging and contamination [46]. In contrast, AFM—particularly tilting-AFM and 3D-AFM—directly measures sidewall topography with sub-nanometer vertical resolution and minimal reliance on modeling. This makes AFM the preferred reference for calibrating CD-SEM contour metrology and for quantifying LER and line width roughness (LWR), especially where electron beam proximity effects and resist shrinkage introduce bias [46].

TEM delivers the highest spatial resolution among all dimensional metrology techniques, which can achieve atomic-scale imaging (about 0.05 nm) with direct visualization of crystal lattices and defects [53,54,55]. It is indispensable for process development, failure analysis, and calibration of reference metrology. For advanced etching processes, TEM enables whole-device imaging of complex three-dimensional structures—such as GAA silicon nanosheets—at moderate resolution while resolving atomic-scale details at specific locations [56]. It also allows analysis of film thickness uniformity and interface integrity in emerging materials, including low-contrast specimens such as carbon nanotubes, graphene, and MoS_2_ [46,57]. Furthermore, when combined with automated FIB systems, TEM supports site-specific CD-TEM and tomography for three-dimensional measurements, while integration with molecular simulations provides insight into defect formation mechanisms [58,59]. However, TEM is inherently destructive, requiring laborious sample cross-sectioning and thinning (typically <100 nm), which precludes its use for in-line monitoring and introduce artifacts. AFM, by contrast, is non-destructive, requires minimal sample preparation, and can scan areas from sub-micrometer to millimeter scale without cutting the wafer.

In summary, AFM occupies a distinct and essential position, bridging the gap between the fast, model-dependent, ensemble-averaged data from optical scatterometry and the ultra-high-resolution, destructive, localized analysis of TEM. Its unique ability to provide direct, traceable 3D topographic information with true sidewall access and minimal sample preparation makes it indispensable for reducing measurement uncertainty in complex 3D IC architectures. The value of these capabilities is ultimately demonstrated in addressing the most demanding real-world challenges—namely, the characterization of ultra-HAR features exemplified by TSVs, and the need to probe surfaces modified by atomic-scale etching processes such as ALE and cryogenic plasma etching. The following sections examine AFM’s application in these critical domains, highlighting both its demonstrated capabilities and ongoing innovations that push the boundaries of nanoscale 3D metrology.

## 3. Characterization of High-Aspect-Ratio Structures: The Through-Silicon Vias

### 3.1. The Metrological Challenges of Deep Reactive Ion Etching (DRIE) for TSVs

The Bosch process, widely adopted for silicon DRIE, relies on the cyclic alternation of two core steps to achieve HAR TSV etching [60,61,62]. Specifically, it involves “alternating deposition of a passivation layer and etching of vias” using C_4_F_8_ (passivation) and SF_6_ (etching) gases, respectively. This cyclic operation enables precise control of anisotropy, while the alternating isotropic dry etching and sidewall passivation/deposition steps inherently form characteristic scallops on the sidewall [60,62]. Compared to conventional etch processes, the Bosch process offers high etch rate and aspect ratio, making it the primary choice for TSV fabrication, but its cyclic nature also introduces multiple metrological challenges for profile control [61].

(1) Etch Depth and Uniformity

Ensuring vias reach the target depth and maintaining depth uniformity across the wafer are a core metrological challenge [63,64]. For 300 mm wafer-scale TSV fabrication, within-wafer depth uniformity as low as ~0.7% is required [65]. Depth variations arise from factors such as inhomogeneous plasma distribution and temperature fluctuations in the etching chamber [64]. From a metrology perspective, these variations must be measured non-destructively across the wafer with sufficient accuracy for process feedback control.

(2) Sidewall Profile

Controlling sidewall taper (positive/negative) and verticality is critical for subsequent liner/barrier deposition and Cu filling [61,62,66]. The SWA is a key geometric parameter, as its deviation from the design value affects step coverage of sputter-deposited layers. Through optimized positive profile etching processes, near-vertical sidewalls can be achieved [66]. The metrological challenge lies in quantifying taper angle deviations and ensuring consistency across the wafer, as non-uniform taper can lead to poor step coverage and filling defects.

(3) Scalloping

Scalloping refers to the periodic ripples on the sidewall induced by alternating Bosch process steps. Quantifying its amplitude and periodicity is essential for process control [60,67]. Scallops are characterized by depth/amplitude and period [68]. These features can cause leakage currents, thermo-mechanical stress, and affect liner/barrier deposition integrity [60]. Metrologically, it is critical to ensure scallop depth remains below acceptable limits (e.g., <80 nm) [66], as large amplitudes degrade device reliability [69].

(4) Bowing/Necking

Bowing/necking refers to deviations from a straight sidewall profile, a common defect in DRIE [65,69]. This phenomenon is quantified by the curvature profile parameter, which describes the extent of sidewall bowing. The metrological challenge is to accurately measure bowing amplitude and spatial distribution, as even slight deviations can affect TSV mechanical stability and electrical performance.

(5) Bottom Profile and Microtrenching

Characterizing bottom rounding, roughness, “grass”, and microtrenching is critical for ensuring TSV reliability. The via bottom often exhibits a curved shape, and the bottom roughness (root-mean-square, rq) typically ranges up to 100 nm [70]. Silicon fin defects (a type of “grass-like” defect) at the bottom are considered “killer defects”, which arise when bulk micro defects in the silicon substrate act as micro-masks during etching and can lead to process or mechanical failures [71]. Microtrenching (bottom corner undercut) and rounding are induced by uneven ion flux and passivation layer distribution during DRIE [62]. From a metrology perspective, key challenges include non-destructive bottom access in high-aspect-ratio vias, sufficient resolution to resolve sub-100 nm microtrenches and fine defects, and the need for chemical or subsurface information to distinguish real defects from normal roughness.

### 3.2. Overcoming the High-Aspect-Ratio Challenge: Innovations in AFM Technology

#### 3.2.1. The Fundamental Limitations of Conventional AFM for HAR Metrology

The application of conventional AFM to the precise metrology of HAR structures like TSVs is fundamentally constrained by a triad of interrelated challenges stemming from the physical probe, the instrument’s architecture, and the operational workflow.

At the most fundamental level, AFM tip–sample convolution effect stands as the primary bottleneck for reliable HAR structures characterization: the finite geometry of standard pyramidal tips prevent them from reaching the bottom or accurately profiling the sidewalls of deep, narrow vias [72]. This results in geometric dilation—an apparent broadening of features—that directly degrades the accuracy of nanoscale width measurements and hinders the acquisition of absolute dimensions [73]. Conventional low-aspect-ratio tips (e.g., 0.6) cause obstructive contacts with adjacent structures, introducing severe artifacts such as broadened planes and merged topography, and ultimately fail to follow the complex contours of steep walls or deep holes in HAR nanostructures [74].

Beyond the probe itself, the scanning and sensing architecture by the instrument can introduce systematic errors. The measurement accuracy is compromised by the nonlinear behavior (hysteresis, creep) of piezoelectric scanners [44,75]. Furthermore, a critical geometrical misalignment, known as Abbe offset, exists when the displacement sensor’s measurement axis is not colinear with the probe tip. During scanning, minuscule angular deviations of the scanner are amplified by this offset, creating Abbe errors that can substantially degrade dimensional accuracy, especially over larger scan ranges [76].

The inherent operational limitations of conventional AFM also hinder its practical utility for HAR samples. The serial nature of scanning results in notoriously low throughput, making the acquisition of statistically significant data or large-area maps impractically slow (minutes to hours per image) and exacerbating the impact of thermal drift [44,75]. This low speed, coupled with complex operation and extensive offline data analysis, prevents standard AFM from functioning as a high-efficiency tool for process development or in-line monitoring in high-volume manufacturing environments [26,77,78].

To overcome these limitations and enable reliable, high-precision HAR metrology, significant innovations have been developed across three fronts: the design of specialized HAR probes, the creation of advanced instrumentation algorithms for true 3D profiling, and the evolution toward fully automated, high-speed inline metrology systems.

#### 3.2.2. Innovation 1: High-Aspect-Ratio Probes (HARPs) for Enhanced Access and Accuracy

**Figure 4:**
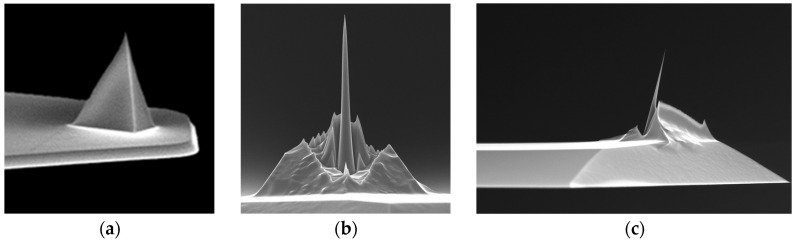
Representative commercial AFM probes: Standard vs. Innovations. (**a**) Ultra-sharp probe (Bruker’s SNL-10 probe, Billerica, MA, USA) designed for high-performance imaging; (**b**) High-Aspect-Ratio (HAR) spike probe (Bruker’s TESPA-HAR probe, aspect ratio 5:1) ideal for semiconductor trench imaging; (**c**) Focused Ion Beam (FIB)-milled probe (Bruker’s FIB2-100S probe, aspect ratio, 20:1) for deep trench measurement. Images adapted from Bruker AFM Probes online catalog [80].

To directly address the tip–sample convolution bottleneck, a variety of HAR probe designs have been developed to enhance access to deep trenches and improve measurement fidelity. As illustrated in Figure 4, commercially available solutions range from ultra-sharp probes to HAR spike probes (aspect ratio 5:1), and FIB-milled probes with aspect ratios up to 20:1, specially designed for deep trench measurement. To achieve even higher aspect ratios, a deformation-suppressed lateral FIB milling strategy enables the fabrication of exchangeable HAR AFM tips with ultra-high aspect ratios (up to 45) and tip diameters as small as 9 nm, overcoming beam-induced deformation through a “smart milling” algorithm that maintains mechanical connection to the frame until the final milling step [79].

The effectiveness of such probes is contingent not only on their geometric design but also on specialized scanning modes. For example, Bruker’s DTSense^TM^ scan mode—integrating adaptive vectored motion and piconewton force feedback—when paired with a 10 nm wide, 200 nm long cylindrical BNT-10-200 probe, enables measurements of HAR trenches in gate-all-around (GAA) devices, achieving gate pitches as small as 44 nm and aspect ratios greater than 8:1. As a non-resonant scan mode, DTSens^TM^ minimizes the probe’s interaction volume, ensuring that the effective probe size remains close to its physical dimensions. This allows it to measure spaces much smaller than those accessible with conventional resonant modes [81].

Even the most advanced probe designs and scanning modes cannot guarantee measurement accuracy without reliable tip characterization and calibration. The geometric parameters of HAR probes—such as tip width, effective length, and sidewall angle—directly influence the fidelity of depth and width measurements, yet these parameters can deviate from nominal values due to manufacturing variability or wear during scanning. To address this, a multi-feature comb-shaped characterizer (linewidths: 15–60 nm, line spaces: 10–50 nm) integrated with nanoscale gratings (pitch uniformity: 0.75%) provides a robust calibration solution. This characterizer extracts key tip parameters (width, effective length, vertical edge height) with consistency within 1 nm compared to conventional methods, providing SI-traceable lateral scale calibration. Multiple lines and spaces of the comb characterizer capture several tip/feature profiles, allowing for stable and consistent extraction of tip parameters by aggregating independent information from individual features [73].

#### 3.2.3. Innovation 2: Advanced Instrumentation and Algorithms for True 3D Profiling

While HAR probes improve access, advanced instrument design and control algorithms are essential for generating accurate, quantitative 3D data from HAR structures.

For true HAR structures with near-vertical sidewalls, a metrological tilting-AFM has been developed, which offers SI-traceable dimensional measurements by controlling the tilting-tip with an inclined servo axis. This technique achieves high sampling density (≥1 nm^−2^) along the line pattern, enabling detailed analysis of LER distribution with sub-0.5 nm profile resolution and 0.07 nm measurement reproducibility [82]. This capability directly addresses the challenge of quantifying sidewall scalloping and roughness.

To address fundamental instrument limitations, such as Abbe offset inherent in some tilting-AFM designs, a post-processing correction of Abbe error has been developed. This approach measures the scanner’s angular error (up to 0.057″ peak-to-valley for pitch angle over 8 µm scans) using a triple-beam laser interferometer and applies a corresponding correction to the AFM data. The effectiveness of this method was demonstrated by a significant improvement in measurement accuracy: the measured pitch of a 1D grating was corrected from 100.12 nm to 99.94 nm, with an expanded uncertainty of 0.05 nm, closely matching reference values obtained from a metrological AFM with negligible Abbe error [76].

**Figure 5:**
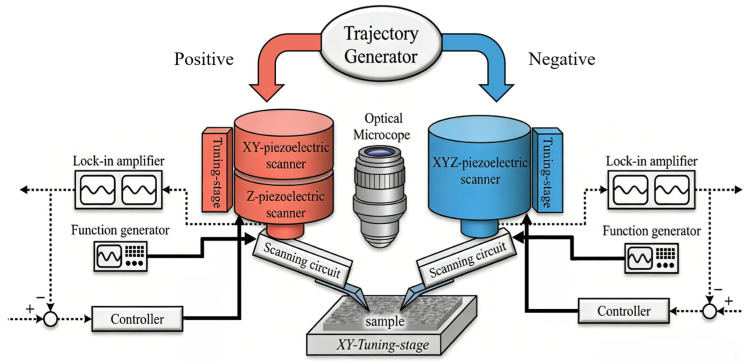
Schematic of the dual-probe AFM system, which consists of two independent scanning units, Akiyama self-sensing probes, an optical microscope alignment module, and high-precision piezoelectric scanners, enabling high-precision 3D topography scanning of steep sidewall structures. Adapted from Ref. [84] (originally proposed by Jim-Wei Wu et al.).

Complementing these hardware innovations, advanced control and data processing algorithms play a critical role in ensuring measurement fidelity. For instance, in systems designed for high-precision sidewall imaging, a dual-probe AFM configurations with adaptive tilting angle algorithms have been proposed and demonstrated in Figure 5. This system can estimate the most effective tilting angle for each probe, allowing tilting angle adjustments after each line scan to handle samples with unknown or varying sidewall angles and obtain high-precision images in a single scan [83]. A neural network complementary sliding mode controller further compensates for xy-piezoelectric scanner hysteresis and resists external disturbances, ensuring precise tracking of scanning trajectories [84]. Such algorithms, combined with slope-based merging techniques, can reduce SWA error from 27.3% to 4.5% for standard gratings by stitching results from two probes with different tilting angles and ensuring precise probe alignment [84].

#### 3.2.4. Innovation 3: Automated Inline AFM Systems High-Throughput Metrology

To address the diverse inline metrology requirements for advanced semiconductor manufacturing—ranging from EUV lithography process control to high-throughput wafer inspection—several innovative AFM systems have been developed. Each system targets specific process nodes and metrology challenges, as detailed below.

- QUADRA: A high-speed inline AFM for EUV lithography beyond 5 nm nodes. The QUADRA system features four parallel independent miniaturized AFM (MAFM) heads, high-bandwidth miniaturized z-scanners, and a proprietary Feed Forward Trajectory Planning (FFTP) scan mode. This design enables non-destructive measurement of deep and narrow photoresist patterns at unprecedented speed (10–100 WPH) and noise floor (50 pm) [77]. On-tool automatic data processing extracts CDs (depth, width), LWR, LER, and sidewall parameters. A contour-based algorithm further discriminates between depth-dependent LER variation and photoresist defects (scum/footing), providing essential feedback for EUV process control [77]. With tip-to-tip matching (TTTM) and pm-range precision, QUADRA meets high-volume manufacturing (HVM) process control needs.
- Automated 3D-AFM: Targeting precise measurement of SWR for 3D structures like FinFET, 3D NAND, and TSV. This fully automated system utilizes a tiltable z-scanner (±38° from perpendicular axis) to access vertical and undercut structures, and an equipment front end module (EFEM) for automatic wafer loading and probe positioning [78]. Equipped with a novel SWR automatic analysis software featuring auto-flattening, sidewall detection, and roughness calculation, the system reduces analysis time to under 2 s per image. Wafer-level (13 dies) measurements achieved a reproducibility relative standard deviation below 2% [85].
- Pattern-centric Inline AFM: for 3D NAND interconnect monitoring. For 3D NAND wafer interconnection process monitoring, a novel pattern-centric inline AFM metrology solution has been proposed. It integrates the Image Explorer for Advanced Metrology (IE-AM) engine to automatically process AFM data, accurately extract Vertical Interconnect Access (VIA) dishing depth and barrier height even for shallow VIA features with insufficient edge contrast [86]. The IE-AM engine integrates with existing fab command systems, uses the design layout as reference to identify VIA features among complex patterns, process large volumes of AFM data, and provides feedback for process control or tool shutdown in case of deterioration [86].
- Parallel Microelectromechanical System (MEMS) AFM Platform: A high-throughput inline AFM platform based on parallel MEMS AFM devices. Each 1 mm^2^ footprint device enables three degrees of freedom (xyz) movement and self-sensing, and a four-device linear array achieves simultaneous scanning. This architecture can be scaled to thousands of devices, enabling full 300-mm wafer coverage in 1 h (vs. 400 days for traditional single-head AFM) [87]. The parallel MEMS AFM platform supports after-etch inspection (AEI) applications, including LER metrology (measuring 5.2 ± 0.46 nm unintentional LER and 12.7 ± 1.09 nm intentional LER on line-space structures) and rapid defect inspection (detecting 60 nm × 150 nm bridge defects in 0.3 s and 100 nm × 150 nm break defects at 64 px × 64 px resolution) [87].

These innovations collectively transform AFM from a slow, laboratory-bound instrument into a viable, high-throughput metrology solution capable of addressing the demanding requirements of modern semiconductor manufacturing.

### 3.3. AFM’s Application in Quantitative TSV Metrology

The technological advances discussed above—spanning specialized HAR probes, tilting-AFM architectures, and automated inline systems—directly address the intrinsic metrology challenges of TSV fabrication. Their practical deployment in TSV process control falls broadly into two categories: direct post-etch profile quantification for process validation, and post-CMP planarity metrology for subsequent integration steps.

#### 3.3.1. Post-Etch Profile Analysis and Process Development Feedback

**Figure 6:**
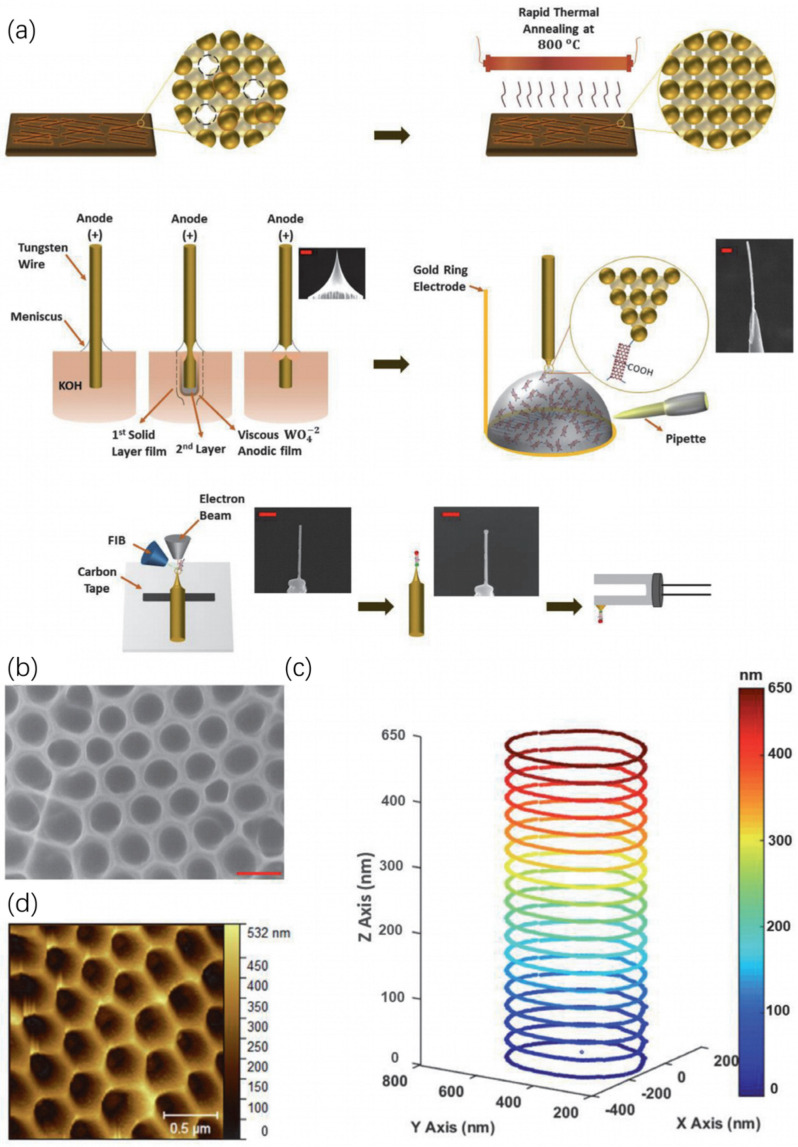
(**a**) Illustration of the probe fabrication; (**b**) SEM image of AAO with the depth 1 μm and pore diameter 350 nm; (**c**) 3D image showing the tip scanning 650 nm deep inside a hole: the tip descends to the bottom (marked dot), moves laterally to locate the sidewall, then follows the boundary in a 360° scan before retracting stepwise to scan the next rim; a straight line in the scan indicates tip exit or completion. (**d**) Raster image obtained from conventional AFM used to find the location of hole. Reproduced from ref. [89] with permission.

AFM’s core advantage lies in its ability to non-destructively quantify critical post-etch TSV parameters (such as TSV top opening CD, upper SWR, and partial depth) with high resolution, supported by HAR probes and optimized scanning algorithms [88]. For instance, a customized HAR multiwall carbon nanotube (MWCNT) AFM probe has been employed to scan the interior topography of anodic aluminum oxide (AAO) structures that mimic TSV geometries. As shown in Figure 6, a custom concentric scanning algorithm guides the tip to descend to the bottom of a 350 nm diameter pore and trace the sidewall in rings, achieving a scan depth of ~650 nm and enabling SWR analysis that conventional AFM cannot provide [89]. This direct topographical data of TSV is essential for evaluating etch process uniformity, and the variations in top opening CD or SWR can indicate non-uniform plasma etching effects [90].

AFM’s quantitative feedback is invaluable for process optimization. Studies on DRIE-fabricated silicon TSVs reveal that SWR varies significantly with vertical depth—the upper sidewall (50 μm depth) exhibits a much higher average roughness ($R_{a}$) of 438.61 nm, while the lower section (400 μm depth) shows a markedly reduced $R_{a}$ of 151.11 nm—a difference attributed to the upper region experiencing more alternating etching-passivation cycles during the Bosch process [91]. This depth-dependent roughness information, captured directly by AFM, provides quantitative insight for tuning DRIE parameters to achieve more uniform sidewall quality.

Furthermore, under the framework of hybrid metrology, 3D-AFM serves as a critical “ground truth” reference. For instance, AFM-derived priors can be integrated into optical models such as optical critical dimension (OCD) scatterometry using Bayesian methods to reduce measurement uncertainty on inaccessible features. Zhang et al. [92] explicitly demonstrated that AFM measurements on accessible reference structures (e.g., top width, middle width, height) provide high-accuracy values with known uncertainties, from which Gaussian prior distributions are constructed. When fitting the optical model to scattering data from inaccessible structures (e.g., sub-40 nm trenches), the posterior distribution combines the likelihood from optical signals with these AFM-derived priors, effectively constraining the solution space and breaking parametric correlations that otherwise plague standalone OCD regression [92,93]. Quantitatively, this Bayesian hybrid approach reduced the top-width uncertainty from 10.8 nm (OCD alone) to 0.9 nm, and the middle-width uncertainty from 0.4 nm to 0.2 nm [92]. More broadly, hybridizing OCD with AFM can reduce top-width uncertainty by up to 4 nm, and total measurement uncertainty (TMU) values below 0.2 nm have been reported for model-assisted extrapolation [46]. As a related example in the broader context of Bayesian hybrid metrology, Rana et al. [85] used Bayesian learning of neural network weights with CD-AFM reference data, achieving a TMU of 0.00 nm (3σ) for sub-40 nm trenches—a significant improvement over simple extrapolation errors > 2 nm. Thus, embedding AFM priors via Bayesian methods—whether in optical regression or machine-learning framework—enables accurate CD prediction in the capability-limited regime where no single technique meets the required sub-0.5 nm uncertainty. This demonstrates AFM’s indispensable role in validating and refining the faster optical techniques used for inline process monitoring.

#### 3.3.2. Post-Chemical Mechanical Polishing (CMP) Planarity Management

While the focus of this review is etching metrology, it is worth noting that AFM also plays a critical role in subsequent TSV process steps, such as CMP. AFM enables quantitative measurement of post-CMP dishing and erosion in trench structures, with its high lateral and vertical resolution allowing for detailed visualization of surface morphology at the nanoscale [94]. For example, in TSV CMP process development at the 28 nm technology node, AFM can accurately track the progressive reduction of Cu from ~330 Å to 110 Å during multi-step polishing, and verify that pre-CMP annealing suppresses Cu extrusion from over 500 Å to approximately 60 Å [95]. This quantitative feedback, combined with AFM’s ability to resolve post-CMP root mean square (RMS) surface roughness down to 1.8 nm [96], provides the critical process insight required to achieve the high planarity essential for successful 3D stacking.

### 3.4. AFM in the Context of TSV Metrology: A Comparative Assessment

In semiconductor manufacturing, SEM, optical metrology, and stylus profilometry each play important roles. For TSV metrology, however, AFM occupies a distinct and indispensable place. A comparative assessment of these techniques reveals not direct competition but complementary strengths, with AFM addressing characterization challenges that other methods cannot.

#### 3.4.1. AFM vs. SEM: Complementary Roles for Precision and Throughput

CD-SEM is widely adopted for inline TSV-related dimensional metrology due to its high throughput and automation, providing rapid top-down imaging of full TSV profiles for process monitoring [82,97]. However, its fundamentally 2D nature limits true 3D characterization: sidewall profiles quantification is indirect, and roughness measurements are compromised by the “edge effect” and random noise in the high-frequency region, especially at the sub-nm scale [82,97,98]. As semiconductor devices continue to shrink and adopt increasingly complex 3D geometries, these limitations render CD-SEM insufficient for precise TSV sidewall metrology.

In contrast, AFM offers non-destructive, SI-traceable quantitative 3D data for TSV sidewalls—a key advantage for critical TSV characterization where sample integrity and dimensional accuracy are paramount [82,99]. Specifically, tilting-mAFM achieves sub-nm resolution (<0.5 nm in the sidewall scanning direction) and high reproducibility (0.07 nm standard deviation of LER) with a high sampling density (≥1 nm^−2^), enabling analysis of LER distribution along the TSV height [82,99]. Direct comparison of SEM and tilting-AFM measurements at identical TSV-relevant line pattern locations shows that SEM overestimates LER (4.94 nm vs. 4.53 nm for AFM) and correlation length (48.8 nm vs. 42.6 nm), while AFM reliably extracts the full set of roughness parameters (σ = 1.64 nm, α = 0.87, ξ = 42.6 nm) via PSD, height-height correlation function (HHCF), and autocorrelation function analysis [97,100]. Furthermore, PSD analysis reveals that AFM’s high-frequency noise is several orders of magnitude lower than that of typical SEM methods, ensuring more accurate capture of fine TSV surface roughness [82]. This superior fidelity stems from AFM’s vector probing approach, which minimizes tip wear and realizes detailed TSV sidewall mapping from inclined directions, avoiding the edge-detection ambiguity inherent in SEM’s secondary electron imaging [98].

Recent advances have substantially mitigated AFM’s traditional low-throughput bottleneck. HS-AFM scan-heads with separated xy- and z-scanners and photothermal cantilever excitation achieve imaging rates up to 1.9 kHz (constant height mode, 1 µm × 1 µm scan, 100 × 100 pixels), while the Noise2Noise machine learning algorithm reconstructs high-resolution TSV-related AFM images from fast-scan noisy data in seconds—reducing processing time by up to 500× compared to manual processing [99,101]. Interferometric direct height measurement (vertical resolution < 0.1 nm) further decouples topography measurement from feedback loop constraints, enabling 1 frame/s imaging of areas up to 36 × 36 µm^2^ with sub-nm accuracy for in-line TSV process control [102]. Concurrently, the multi-feature comb-shaped characterizers enable precise tip geometry calibration, reducing a key source of uncertainty in HAR feature metrology [73]. Despite these advances, inherent limitations persist. AFM probes cannot penetrate dense TSV arrays, and tip geometry and interaction forces constrain HAR characterization. In particular, bifurcation effects in AFM amplitude-phase signals when probing steep TSV sidewalls introduce nanometer-scale uncertainties that require advanced data processing to resolve.

In industrial practice, SEM and AFM are not mutually exclusive but rather serve complementary roles. SEM provides rapid qualitative screening and large-area coverage as an inline monitoring tool, while AFM functions as a reference metrology for calibrating SEM LER measurements, validating 3D TSV profiles, and delivering traceable quantitative data for process qualification and tool matching. Recent developments further reinforce this complementary paradigm: a hybrid positioning system integrating SEM and AFM has demonstrated reproducible sample positioning accuracy of 6.5 ± 2.1 μm (x-axis) and 4.5 ± 1.7 μm (y-axis) across 12 repeated measurements, enabling precise multi-modal characterization of the same region of interest without sample transfer—a critical capability for correlative TSV metrology in R&D and failure analysis [103]. Moreover, in hybrid bonding applications, a quantitative correlation between in-line SEM (using directional back-scattered electron imaging) and AFM reference measurements has been established, achieving root mean square errors of 0.42 nm for 500 nm Cu pads and 0.47 nm for 350 nm Cu pads—demonstrating that SEM can be calibrated against AFM to deliver production-worthy nanotopography metrology while maintaining high throughput [104]. This hybrid strategy addresses the metrological challenges of advanced TSV structures with shrinking dimensions and complex 3D geometries, balancing throughput and precision for both in-line inspection and off-line reference calibration.

#### 3.4.2. AFM vs. Optical Metrology: Direct Measurement vs. Model-Dependent Inference

Optical metrology techniques (e.g., scatterometry, reflectometry) are the mainstream choices for inline process control in the semiconductor industry due to their core advantages of high throughput and non-destructiveness [26,105]. However, these are inherently indirect measurements that rely on complex modeling analysis and require prior knowledge of the sample surface, and discrepancies in the modeling may lead to inaccurate measurement results [26].For emerging 3D nanostructures (e.g., 3D stacked memories, FinFET devices) or process excursion scenarios, optical metrology techniques often struggle to adapt, as their models are difficult to cover random variations in the sample’s optical properties, film thickness, etc., thereby affecting measurement reliability.

AFM’s role here is as a crucial “model validator” and “uncertainty reducer” [26,105]. In industrial practice, AFM reference data are routinely used to build and calibrate OCD model libraries—a critical step before deploying optical tools for inline monitoring. Specifically, AFM provides high-fidelity 3D topographic datasets that serve as the basis for extracting spectral transfer characteristics and correcting model discrepancies in scatterometry [75]. By acquiring accurate 3D topographic reference datasets of samples using AFM and performing correlation analysis with measurement data from optical metrology tools, key parameters such as the spectral transfer characteristics of optical tools can be comprehensively characterized, and model discrepancies can be corrected [75].

In practical applications, AFM has been successfully used to verify the accuracy of OCD models, providing indispensable support for the reliable application of optical metrology techniques in deep sub-micron processes [105]. Thus, while optical metrology dominates inline production due to speed, AFM plays an essential role in model establishment, periodic verification, and root-cause analysis when process deviations occur—a complementary relationship that ensures both throughput and measurement fidelity in TSV manufacturing.

#### 3.4.3. AFM vs. Stylus Profilometry: Resolution for Nano- vs. Micro-Scale Features

AFM and stylus profilometry are two widely used contact-based metrology techniques addressing different scales of feature measurement for TSV [40,106]. Stylus profilometry is well suited for measuring deep, macroscopic features such as large steps or overall wafer bow over extended scan ranges [107]. However, its utility for fine-structure characterization is severely constrained by lower lateral resolution and larger tip radii (typically ~1.7–400 μm for commercial styli) [40,107]. This geometric limitation restricts the ability to resolve fine structures, and comparative studies indicate that stylus measurements can underestimate feature dimensions by an average 9% relative to optical methods—a discrepancy attributed to tip wear, limited lateral resolution, and other systematic inaccuracies [106].

These limitations make stylus profilometry unsuitable for narrow, high-density TSV features: TSVs typically have diameters < 10 μm and pitches of tens of microns, requiring precise characterization of narrow gaps and sidewalls [108], but the large stylus tip cannot access narrow spaces or avoid deformation in limited probing space, leading to measurement errors [109]. The incompatibility is further exacerbated by the morphological dilation effect of stylus tips: the measured profile of surface features is the dilated result of the tip geometry, which is particularly problematic for dense, narrow TSV structures [40].

AFM, particularly 3D-AFM and CD-AFM, is the appropriate tool for high-resolution metrology of TSVs [78]. Its small tip size (<50 nm) and specialized flared or HAR tip designs enable access to near-vertical sidewalls and the precise quantification of nanoscale roughness and critical dimensions [110]. Therefore, in practice, stylus profilometry is used for deep, large-scale morphological characterization (e.g., post-grinding TSV exposure), whereas AFM—particularly its CD and tilting variants—is deployed for high-resolution sidewall analysis in R&D, process development, and inline reference metrology where nanoscale precision is required.

## 4. AFM in the Realm of Atomic-Scale Etching Processes

The ultimate frontier for 3D IC fabrication lies in achieving atomic-scale precision and minimal damage. Advanced etching techniques, notably ALE and cryogenic plasma etching, have emerged to meet these demands. This section examines how AFM-based metrology has been indispensable in developing, validating, and refining these processes, moving beyond mere characterization to become a critical enabler of atomic-scale manufacturing.

### 4.1. Atomic Layer Etching: Characterizing the Ultimate Precision

#### 4.1.1. Introduction to ALE

ALE is a cyclic process that achieves material removal with atomic-level control by separating the etch into two self-limited steps: surface modification and subsequent removal of the modified layer [111,112]. This self-limiting nature is the key differentiator from continuous-wave plasma etching, as the etch depth saturates with process time and depends solely on the number of cycles, enabling exceptional uniformity and control [111,113,114].

ALE’s precision has been demonstrated across a wide range of materials. For metal oxides, Al_2_O_3_ ALE uses alternating hexafluoroacetylacetone (Hhfac) and H_2_ plasma for Al_2_O_3_ and halogen-free N_2_/O_2_ plasma for HfO_2_, enabling controlled EPC and significant surface smoothing [115]. For 4H-SiC, bias-pulsed ALE using Ar/Cl_2_ plasma has realized sub-angstrom surface roughness (0.83 ± 0.08 Å) and a high ALE synergy of 99% [116]. Controllable etch rates further reflect ALE’s precision: O_2_/BCl_3_-based ALE for AlGaN achieves 0.68 nm/cycle [117]; BCl_3_/Ar-based ALE for GaN yields ~0.73 nm/cycle [118]; and thermal ALE for Mo via sequential O_3_ and SOCl_2_ exposures exhibits etch rates ranging from 0.94 Å/cycle to 10.98 Å/cycle depending on temperature [119]. This level of control is paramount for next-generation semiconductor devices. Scaled FinFETs and GAA FETs feature densely arranged HAR structures and sub-10 nm critical dimensions, imposing stringent requirements on etching accuracy, conformality, and selectivity [112,114]. Traditional etching techniques struggle to meet these requirements, while ALE, with its atomic-scale precision, low damage, and conformality, can precisely control key steps such as channel recess depth and spacer etching, while ensuring material selectivity, making it a core technology supporting the fabrication of these advanced devices [112,113,114].

However, this atomic-scale precision introduces a significant metrology challenge: how to reliably measure and confirm processes that manipulate materials a few atomic layers at a time. This is precisely where AFM’s unique capabilities become essential.

#### 4.1.2. AFM’s Role in ALE Process Development and Validation

The realization of ALE hinges on the precise control and validation of three fundamental, interrelated characteristics: (i) self-limiting saturation, where etch depth is governed by cycle count rather than process time; (ii) surface smoothing, arising from preferential removal of protruding features; and (iii) minimal subsurface damage, a direct consequence of confining reactions to a modified surface monolayer. While in situ metrology such as spectroscopic ellipsometry provides valuable real-time tracking of bulk film thickness removal with high throughput, it operates on an optical model-based, macroscopic average. Conversely, high-resolution TEM offers atomic-scale visualization of lattice structure but is inherently destructive, statistically limited by small sampling areas, and ill-suited for quantifying the surface topography evolution during cyclic processing. Bridging this critical characterization gap is AFM.

AFM is uniquely positioned as the indispensable metrology tool for ALE development because it provides direct, local topographic mapping with sub-angstrom vertical resolution, enabling the quantitative decoupling of lateral and vertical etch components and the verification of near-atomic flatness without altering the fragile surface chemistry. The following sections examine how AFM enables direct validation of each ALE metric, from establishing fundamental etch rates to verifying final surface quality and confirming the process’s inherently low-damage nature.

(1) Etch-per-Cycle (EPC) Measurement: Validating Self-Limiting Saturation

The defining characteristic of ALE—self-limiting saturation—is validated through EPC measurement.

AFM accurately determines EPC by measuring the step height of patterned test samples after a known number of ALE cycles, then dividing the step height by the cycle count [116,120,121]. This approach is exemplified in 4H-SiC bias-pulsed ALE, where 20 × 20 μm^2^ AFM scans were used to measure etch depth and EPC was calculated by averaging step heights from positions ≥ 100 μm apart on the patterned sample [116]. A similar AFM-based methodology applied to AlGaN/GaN HBr/Ar ALE yielded material-dependent EPC values of 0.81 Å/cycle for AlGaN and 2.64 Å/cycle for GaN, based on averaged step heights from four sample locations [121]. For SiO_2_ ALE, the use of smaller AFM scans (500 × 500 nm and 1000 × 20 nm) on roughened patterned films was required to reliably extract an EPC of 0.41 ± 0.01 Å/cycle after 300 cycles [120]. Beyond direct EPC measurement, AFM can also be employed in conjunction with other techniques: for HfO_2_ halogen-free ALE (0.23–1.07 Å/cycle) was derived from thickness changes measured by in situ ellipsometry, with AFM verifying surface smoothing effects that support the accuracy of the measurement [122].

Across these material systems, the consistent observation of cycle-dependent—rather than time-dependent—etch depth indicates self-limiting saturation as the defining characteristic of ALE, a fundamental departure from continuous etching techniques. Moreover, precise EPC measurement is critical for fabricating high-performance devices, as it enables controlled etch depth in applications such as SiO_2_ photonic resonators (approaching intrinsic Q limits) and informs the optimization of process parameters including temperature, pressure, and gas flow [116,122].

(2) Surface Roughness and Smoothing Analysis: Verifying Low-Damage Processing

The self-limiting surface reactions in ALE not only enable atomic-level etch depth control but also inherently minimize surface damage—a key advantage over continuous etching techniques such as RIE, which typically induce severe surface roughening via unregulated ion bombardment [116,120,123]. AFM is widely employed to quantify RMS surface roughness before and after ALE processing.

**Figure 7:**
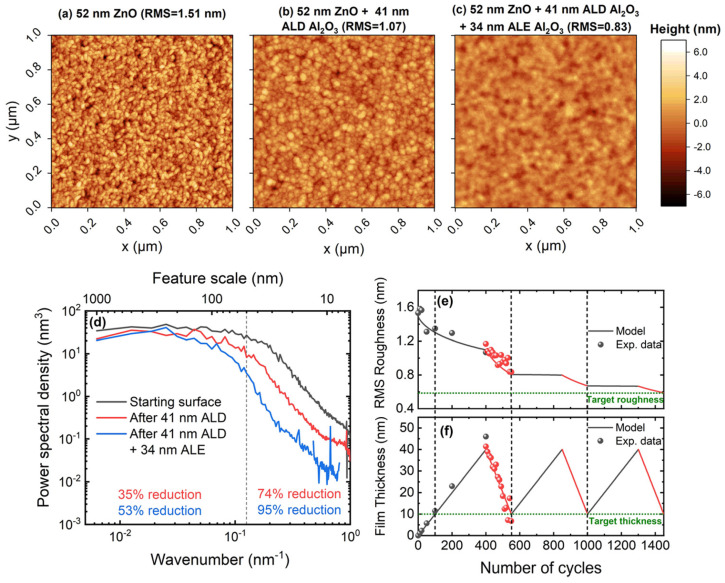
AFM characterization of surface smoothing during Al_2_O_3_ ALE. (**a**–**c**) Height maps showing the evolution from rough ZnO substrate to ALD-coated and ALE-smoothed surfaces. (**d**) Power spectral density (PSD) analysis confirming preferential smoothing of high-frequency (sub-50 nm) roughness features. (**e**,**f**) RMS roughness and film thickness evolution during ALD + ALE processing, with model predictions showing excellent agreement with experimental data. Reproduced from ref. [123] with permission.

**Figure 8:**
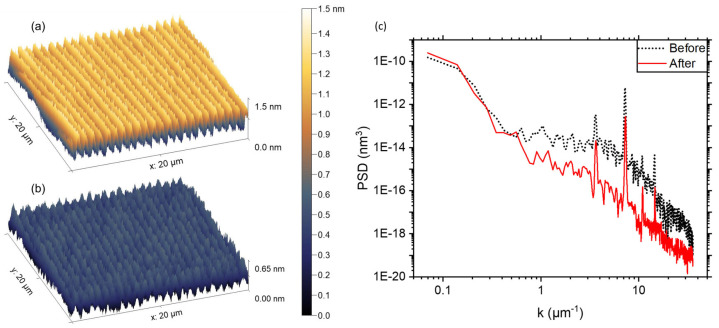
AFM characterization of 4H-SiC surface smoothing by bias-pulsed ALE. (**a**) As-received surface before etching; (**b**) Surface after 200 ALE cycles, showing subangstrom roughness (0.83 ± 0.08 Å); (**c**) PSD analysis comparing the surface before and after etching, demonstrating effective smoothing across all spatial frequencies. Reproduced from ref. [116] with permission.

AFM measurements confirm ALE’s material-specific smoothing efficacy across a range of technologically relevant materials. For Al_2_O_3_ RMS roughness decreases from 1.17 ± 0.07 nm to 0.83 ± 0.07 nm after 150 cycles (see Figure 7) [123]; for SiO_2_, from 3.49 nm to 1.33 nm (a 62% reduction) after 300 cycles [120]; and for 4H-SiC, from 4.91 ± 0.24 Å to 0.83 ± 0.08 Å (subangstrom level) after 200 cycles (see Figure 8) [116]. Similar smoothing behavior is observed in MgO-doped LiNbO_3_ (LN) from 2.07 ± 1.16 nm to 0.34 ± 0.07 nm (an 84% reduction) after 50 cycles [124], and in halogen-free HfO_2_, where roughness is reduced from 1.41 nm to 0.57 nm after 20 cycles [122].

These results reflect material-specific mechanisms (e.g., curvature-dependent fluorination, high volatile byproducts) and confirm that ALE’s self-limiting reactions preferentially remove protruding features while preserving valleys. This enables a degree of atomic-scale planarization unattainable by continuous etching techniques such as RIE.

(3) Damage Assessment: AFM as a Unique Tool for Low-Damage Validation

A critical advantage of ALE over conventional etching techniques is its ability to minimize subsurface damage—a direct consequence of confining reactions to the modified surface layer. Validating this low-damage nature requires angstrom-level morphology characterization, a capability that most conventional techniques lack [125]. Aberration-corrected TEM, for instance, offers sub-angstrom spatial resolution but causes substantial sample damage and requires harsh conditions; meanwhile, electron energy loss spectroscopy (EELS) and atom probe tomography (APT) focus on chemical or compositional analysis rather than direct morphology characterization.

AFM uniquely addresses this gap by enabling angstrom-level morphology characterization with minimal sample damage. Its lateral resolution of ~0.1 nm (1 Å) and vertical resolution of 0.1 pm (0.001 Å) are compatible with various surfaces and environments. This capability is irreplaceable because angstrom-scale surface roughness directly degrades the performance of ultrathin semiconductor and photonic device—precisely the application space where ALE’s low-damage promise is most critical.

Experimental results further underscore AFM’s critical role in validating ALE’s low damage nature, which stems directly from the self-limiting surface reactions. For example, HfO_2_ roughness decreases by 60% (1.41 → 0.57 nm) after 20 ALE cycles [122]; 4H-SiC roughness reaches a subangstrom level of 0.83 ± 0.08 Å (subangstrom level); and MgO-doped LN maintains a roughness of 2.5 ± 0.3 Å at 200 °C, consistent with unetched samples. AFM is thus explicitly highlighted as a core technique for atomic-scale surface morphology analysis, with low sample damage and broad versatility serving as its key advantages.

In summary, ALE’s ultimate precision is confirmed by angstrom-scale EPC across diverse materials, directly validating its self-limiting saturation behavior. Its low-damage nature—a defining advantage over continuous etching—is uniquely validated by AFM, a capability unmatched by other techniques. The angstrom-level surface integrity revealed by AFM confirms ALE’s core promise: minimizing lattice damage inherent to high-energy ion bombardment in conventional plasma etching. This is a critical requirement for next-generation devices where ultrathin layers demand defect-free interfaces.

### 4.2. Cryogenic Plasma Etching: Probing Surfaces at Low Temperatures

#### 4.2.1. Introduction to Cryogenic Etching

Cryogenic Plasma Etching has emerged as a key technology for deep material etching in microfabrication, particularly excelling in the fabrication of HAR structures, and it is gradually replacing traditional anisotropic wet chemical etching processes [126]. Its core principle lies in cooling the substrate to extremely low temperatures (typically below −80 °C) using liquid nitrogen, which promotes the formation of a stable passivation layer from etch byproducts on the material sidewalls while inhibiting the attack of etchant species on the passivation layer, thereby achieving highly anisotropic etch profiles [126,127,128]. The formation mechanism of the passivation layer varies depending on the etching target: when etching silicon, the SF_6_/O_2_ plasma system generates a SiO_x_F_y_ passivation film; when etching SiO_2_, C_4_F_8_ gas is physisorbed on the substrate surface at low temperatures to form a fluorocarbon-like passivation layer, followed by argon plasma to activate the etching reaction [126,127].

The low-temperature environment not only enhances the stability of the passivation layer but also reduces the etch rate of the mask material, enabling cryogenic etching to achieve higher mask selectivity than the Bosch process (selectivity to photoresist > 100:1 and up to 1000:1 for oxide masks). Unlike the Bosch process, which exhibits SWR due to alternating etching-deposition steps, the non-switching nature of cryogenic etching results in much smoother sidewalls, an advantage that is particularly significant in advanced semiconductor devices and MEMS structures [126]. In terms of etch rate, cryogenic etching outperforms the Bosch process in through-wafer etching applications, offering higher efficiency in fabricating deep-through structures. Notably, cryogenic etching processes demonstrate excellent process stability with minimal reactor wall contamination, effectively avoiding process drift even after multiple etching cycles [127,128]. For the etching of SiO_2_, the cryogenic atomic layer etching (cryo-ALE) process optimized by adjusting parameters such as temperature, pressure, and purge time can achieve highly linear etching over 150 cycles, with an etch per cycle as low as 0.13 nm/cycle, meeting the precision requirements of nanoscale device fabrication [128].

Cryogenic etching offers three distinct advantages: the absence of scalloping due to its non-switching passivation mechanism, the ability to fabricate ultra-smooth HAR structures, and the compatibility with temperature-sensitive device layers. Validating these capabilities demands a metrology approach focused on quantifying sidewall smoothness, verifying HAR profile fidelity, and correlating process parameters with these unique structural outcomes. AFM, particularly in advanced 3D configurations, provides the necessary metrology to assess each of these defining features, as elaborated in the following section.

#### 4.2.2. AFM for Post-Cryo-Etch Diagnostics: Validating Smoothness, Profile Fidelity, and Process Stability

In the characterization of cryogenic plasma etching, AFM serves as one of the core tools for acquiring key parameters of nanostructures, such as height and surface roughness [129,130]. These measurement capabilities directly address the unique advantages and associated challenges of the cryogenic etching process.

- Validating the scallop-free advantage. The most distinctive feature of cryogenic etching—its non-switching continuous passivation mechanism—eliminates the periodic scalloping inherent to the Bosch process. AFM provides the direct, quantitative proof required to validate this advantage. By performing SWR measurements on cleaved samples or using specialized tilting-AFM, researchers can directly compare the SWR of cryo-etched features against those from a Bosch process [131]. An automated 3D-AFM study on silicon sidewalls imaged at an optimized tilt angle (e.g., 45°) can reveal an RMS roughness for a cryo-etched sidewall that is significantly lower and lacks the characteristic periodic signature of a Bosch-etched sidewall [132]. This capability is not merely a metrological metric; it provides direct confirmation that cryo-etch eliminates scalloping—a key advantage for HAR structures requiring low-loss optical or electrical performance.
- Assessing profile fidelity in HAR structures. The ability to fabricate HAR features with vertical sidewalls and minimal undercut is another hallmark of cryogenic etching. AFM provides comprehensive 3D profile analysis essential for verifying this capability. AFM is particularly valuable for ex situ characterization of cryo-etched features, enabling precise quantification of sidewall smoothness and 3D profile analysis of micro- and nanoscale structures [132]. Advanced 3D-AFM systems with dual scanners and vector probing scanning (VPS) schemes address conventional AFM limitations, allowing access to steep sidewalls, overhangs, and undercuts with controllable scanning density [132]. For cryo-etched samples with smoother sidewalls, ex situ (off-line) AFM is preferred over in-line CD-mode AFM, as the latter is constrained by noise floor and tip shape limitations [131]. Novel probe designs, such as slender optical fiber probes (OFP) with tiltable holders (−45° to 45°) or wear-resistant CD tips, further enhance access and measurement fidelity in these challenging HAR geometries [132,133]. Collectively, these AFM capabilities ensure that the verticality and smoothness promised by cryogenic etching are accurately verified.
- Enabling process optimization through quantitative feedback. Beyond post-etch verification, AFM provides the quantitative feedback necessary to correlate process parameters with etch outcomes—a prerequisite for establishing robust cryo-etch processes. Systematic AFM characterization of Si nanowires etched under controlled variations of O_2_ concentration and ICP power has quantitatively mapped the dependence of surface roughness and aspect ratio on process parameters, which is a critical step toward establishing a quantitative correlation between AFM topography and cryo-etch conditions [129]. This capability directly supports the optimization of cryogenic etching parameters to achieve target specifications for surface quality and structural fidelity.

In summary, the AFM-based metrology framework described above—from scallop quantification and sidewall profile reconstruction to process parameter correlation—directly addresses the three unique characteristics of cryogenic etching. By providing sub-nanometer roughness verification, comprehensive 3D profile fidelity assessment, and quantitative feedback for process tuning, AFM establishes itself as an indispensable diagnostic tool for the development and optimization of cryogenic plasma etching processes.

### 4.3. Comprehensive Etching-Induced Damage Assessment

Validating the true efficacy of atomic-scale etching processes hinges on a clear understanding of the damage they are designed to eliminate. Conventional plasma etching leaves a complex legacy—surface roughening, crystallographic defects, atomic implantation, and amorphization—against which the performance of advanced techniques must be measured. Characterizing this multifaceted damage landscape demands more than any single technique can offer. It is here that advanced correlative microscopy, with AFM as a central pillar, comes into play, integrating surface topography with subsurface structural and chemical insights to provide the metrological framework essential for confirming whether next-generation etching technologies truly deliver on their promise of atomic precision.

#### 4.3.1. The Nature of Plasma-Induced Damage

Plasma etching-induced damage is not limited to surface roughness but also includes crystallographic defects (e.g., vacancies, displacements, dangling bonds), atomic implantation, amorphization, and carbon depletion [134].

Crystallographic defects like nitrogen vacancies and dangling bonds, mainly induced by high-energy ion bombardment, degrade the electrical properties of materials—for instance, nitrogen vacancies in AlGaN/GaN HEMTs introduce donor-like states and impair interface quality [135]. Atomic implantation is prominent in fluorocarbon plasma etching, where ions such as F^+^ and Ar^+^ penetrate the substrate surface, triggering lattice mixing and changes in bonding states [134,136]. Amorphization and carbon depletion, caused by vacuum ultraviolet radiation and ion bombardment, destroy the original crystal structure—for example, Si–C bond cleavage in low-k materials forms a densified Si–O–Si network [137].

AFM is a core tool for characterizing surface roughness and macro morphology, effectively reflecting the impact of etching on surface flatness, but it cannot detect deep-level damage such as crystallographic defects, atomic implantation, or amorphization [134,135].

Comprehensive damage assessment requires a combination of multiple techniques: TEM for observing crystal structure and amorphization, XPS for analyzing chemical bonds and implanted species, and electrical tests for reflecting the impact of defects on performance.

#### 4.3.2. Correlative Approaches

Correlative microscopy has emerged as indispensable for comprehensive etching-induced damage assessment, as the combination of AFM with TEM, SEM, or FIB milling integrates surface topographic data and subsurface structural/chemical insights [138]. A typical workflow involves AFM locating specific defects or high-roughness areas, followed by targeted TEM or subsurface probe analysis to unravel the full damage extent [139].

The effectiveness of this workflow relies on AFM’s capability to serve as both a precise locator and a quantitative damage assessor. HS-AFM, with nanometric lateral and sub-nanometric height resolution, facilitates real-time imaging of dynamic nanoscale etching events (e.g., pit formation, grain boundary dissolution) and accurate damage dimension measurement [140]. AFM’s quantitative characterization of etching-induced damage (e.g., pit volume, surface roughness) achieves accuracy comparable to TEM, ensuring reliable damage quantification [139].

To translate this capability into practical correlative studies, hardware compatibility must be addressed. Specialized sample holders resolve cross-technique compatibility issues, enabling repeated AFM-TEM imaging of the exact same area without specimen damage [140]. For typical TEM samples (e.g., thin membranes, lamellae), tailored AFM strategies—optimizing cantilever dimensions, adopting tapping mode, or fabricating FEBID tips—overcome surface distortion and accessibility challenges, enabling damage-free correlative imaging without additional preparation [138].

A concrete example illustrates how AFM can serve not merely as a characterization tool but as a precision guide for subsurface analysis [141]. In this workflow, AFM first identifies a sub-100 nm trench defect on an InGaN/GaN structure. A cross-shaped Pt marker is then deposited by electron-beam-induced deposition directly above the region of interest, with arms oriented at 45° to the intended lamella axis to enable sidewall visualization during subsequent FIB thinning. After depositing a protective Pt strap and extracting the lamella via in situ lift-out, the marker appears as two dark features on the sidewalls during ion-beam thinning; their half-spacing directly indicates the distance from the milled surface to the target. By alternately thinning both sides and monitoring marker convergence, the region of interest is precisely centered within the final lamella, enabling direct STEM correlation of AFM surface topography with subsurface damage and structural integrity. Critically, this approach provides real-time positional feedback without introducing additional milling artifacts, eliminating the blind spot inherent in conventional destructive workflows.

The above AFM-guided TEM preparation strategy demonstrates how surface localization by AFM can be effectively combined with subsurface interrogation. This complementarity is also evident in the correlative use of AFM, SEM, and FIB: while AFM excels at high-resolution surface characterization, SEM and FIB milling complement it by revealing subsurface damage. FIB milling uncovers that surface intergranular etching pits often connect to larger subsurface voids formed by intergranular attack, while SEM confirms intergranular/intragranular damage extent [142].

Although seminal correlative microscopy studies were initially demonstrated on model systems such as metallic corrosion and precipitates [101,139,140,142], the underlying principles—precise surface localization followed by subsurface interrogation—are directly transferable to the evaluation of atomic-scale etching processes in semiconductor manufacturing. In the context of 3D chip BEOL processing, this AFM-based integrated approach enables comprehensive assessment of whether advanced techniques like ALE achieve their intended goal: damage-free, atomically precise material removal.

## 5. Grand Challenges, Limitations, and Future Outlook

Despite significant progress, critical challenges and inherent limitations continue to hinder the widespread application of AFM in areas such as TSV profile characterization. Looking forward, addressing these bottlenecks will require transformative technological breakthroughs and the exploration of new research directions to propel AFM toward greater robustness, efficiency, and industrial viability.

### 5.1. The Throughput Barrier: Can AFM Keep Pace with High-Volume Manufacturing?

The fundamental trade-off between resolution and speed remains AFM’s primary limitation, as conventional AFMs typically have a scanning range of around 100 μm and low measurement speed, leading to insufficient throughput for high-volume manufacturing and significant measurement drift over long acquisition times [44,75].

Ongoing hardware innovations address this by developing faster scanner technologies: the ULSS-AFM integrates a compliant nano-manipulator (CNM) to achieve 1 × 1 mm^2^ stitchless characterization, increasing throughput by more than ten times compared with conventional stitched AFMs; the HS Met. LR-AFM combines a nanopositioning and nanomeasuring machine (NMM), piezo stage, and AFM sensor to reach a scanning speed of 1 mm/s, improving calibration efficiency by a factor of 10 while maintaining metrological accuracy [44]. Parallel scanning with multi-probe arrays represents a compelling, though still nascent avenue for throughput enhancement. While this approach theoretically offers a direct path to parallelization, its practical implementation is currently constrained by significant engineering hurdles, including the complicated fabrication of sensor arrays and the associated operational difficulties [44].

Data-driven methodologies complement hardware advancements: the Noise2Noise algorithm reconstructs high-resolution AFM data from fast-scan, noisy, sparsely sampled data, achieving a processing speedup of over 500× and producing outputs comparable to 30-min manual scans [101]. However, such computational approaches face non-trivial challenges, including the lack of a large preexisting AFM image database and limitations of point-to-point targets in conditions where it is impossible to exactly mimic slow, high-resolution scan images from a low-grade source [101]. In contrast, more immediate gains in throughput and accessibility are being realized through software innovations. Open-source analysis platforms like NanoLocz, for instance, demonstrate the power of advanced data processing by integrating trace-retrace intercalation, automatic leveling, and particle tracking algorithms, thereby dramatically reducing manual post-processing efforts and streamlining HS-AFM workflows [143].

Ultimately, the future of high-throughput AFM likely lies in a synergistic combination of robust, manufacturable multi-probe hardware and increasingly intelligent, automated software solutions. This integrated approach is essential for overcoming the inherent resolution-speed trade-off and enabling AFM’s transition from a high-precision lab tool to a practical inline metrology solution for high-volume 3D IC manufacturing.

### 5.2. The Quest for In Situ, Real-Time Monitoring

AFM, as a typical SPM technique, is predominantly an ex situ measurement tool and not suitable for real-time monitoring in reactive plasma environments. When plasma exists in the chamber, the membrane used to isolate the probe and plasma collapses upon contact with the probe, making continuous real-time imaging infeasible [144].

Although optical methods such as optical emission spectroscopy (OES) have emerged as the current preferred choice for real-time plasma monitoring due to their non-invasive in situ sensor and convenient installation [145,146,147,148], they rely on indirect optical signatures and cannot provide the nanoscale topographical and mechanical information that AFM uniquely offers. Thus, the fundamental challenge remains: how can AFM’s rich, direct nanoscale characterization capabilities be extended into the plasma environment?

From a long-term perspective, the development of environment-resistant, MEMS-based “AFM-on-a-chip” sensors holds substantial potential for quasi-real-time feedback through integration into process chambers. Encouraging precedents already exist: a recently developed flexible and thin patch-type plasma resonance spectroscopy probe can be attached to various chamber surfaces (e.g., chuck, chamber wall, bottom) without modifying the chamber design, enabling non-invasive in situ measurement of plasma electron density [149]. Additionally, an in situ real-time dielectric film thickness monitoring system based on planar probes has been validated in commercial Plasma-Enhanced Chemical Vapor Deposition (PECVD) chambers, providing technical support for the integration of MEMS sensors with process equipment [150]. Although these examples are not AFM themselves, they collectively indicate a trend in semiconductor equipment development: the increasing feasibility of embedding compact, resilient sensor systems directly into process chambers for in situ monitoring—a capability that paves the way toward more complex MEMS-based tools, including future environment-resistant AFM probes.

### 5.3. Quantifying and Enhancing Probe Durability: Toward Reliable and Robust AFM Nanometrology

AFM measurement accuracy and repeatability rely heavily on probe durability and precise tip characterization. The development of robust, wear-resistant HAR probes that maintain shape over repeated scans remains a persistent challenge, particularly for industrial applications where tip longevity directly impacts cost and throughput [151].

To address probe wear quantification, several standardized characterization methods have been proposed: a nondestructive method using sharp-structured TipCheck samples was developed in 2024, with estimated tip diameter (ETD) and surface roughness as key metrics, enabling in situ tip morphology assessment without damaging the probe [152]. A purely AFM-based technique was presented in 2018, using sharp spike samples and threshold analysis to measure contact area, allowing rapid calculation of wear volume and material removal rate without specialized equipment [153]. The nanoindentation Hertz model for wear tip radius detection was validated in 2023, with errors within 15%, suitable for spherical or near-spherical worn tips [154].

Optimizing probe durability has been explored through multiple approaches: optimal scanning parameters (200 mV free amplitude, 0.2 Hz scanning frequency, 0.2 set point) were identified in 2024 to minimize wear, enabling 5-h continuous scanning with stable image quality [152]. Hydrophobic alkylsilane self-assembled monolayers (SAMs), particularly FDTS, were shown in 2011 to reduce probe wear by lowering tip–sample adhesion forces, with FDTS-modified probes showing 34% lower wear volume than uncoated silicon probes [155]. HAR-HR probes were developed in 2016 by mounting sharp-tip silver nanowires (AgNW) on cantilevers, achieving aspect ratios of 40:1–70:1 and maintaining stable imaging for 15 continuous scans [151].

For HAR probes specifically, traditional fabrication methods (e.g., FIB/FEB deposition) lead to high costs and poor durability, while AgNW-based probes offer a cost-effective alternative with comparable or superior performance. However, challenges persist, including the chemical stability of AgNWs in air and limited performance in extreme environments [151]. Resolving these issues, alongside achieving scalable fabrication, will be essential for deploying AgNW-based HAR probes in 3D IC manufacturing fabs. Future AFM probe development for industrial HAR metrology must therefore harmonize aspect ratio, durability, and cost.

### 5.4. Future Research Directions: Charting the Next Decade

Despite ongoing efforts to address the key challenges—low throughput, lack of real-time in situ monitoring, and limited probe durability—several fundamental gaps remain. The following four research directions directly address these persistent gaps, building on the solutions already developed.

#### 5.4.1. From Hybrid to Truly Correlative Metrology

Hybrid approaches (e.g., AFM + optical scatterometry) have reduced uncertainty, yet no single technique can simultaneously provide topography, subsurface structure, and chemical information. Complete damage assessment requires combining AFM with TEM or XPS—but typically on different samples. The next frontier is integrating AFM with complementary tools on a single platform for truly correlative analysis.

A core development direction of correlative metrology is integrating AFM with complementary techniques such as SEM onto a single platform, enabling the extraction of correlative, multiparametric, and multidimensional information from samples to achieve the linked characterization of topography with material, chemical, and electrical properties [156]. As a core correlative metrology technique, AFM-in-SEM requires continuous optimization of its in situ workflow to realize accurate in situ correlated measurement of key electrical parameters (e.g., dopant concentration, carrier types) and sample topography in semiconductor materials [157]. The integrated AFM-SEM single-platform system needs to further seamlessly incorporate supporting SEM functional techniques such as FIB, EDX, and SIMS, enriching the dimensions of correlated analysis between material chemical composition, 3D elemental distribution, topography, and electrical properties [156].

Beyond expanding the capabilities of the AFM-SEM system, integrating AFM with other complementary techniques, such as AFM-Raman, represents an equally important frontier in correlative metrology. While AFM-SEM-FIB-EDX workflows excel at structural, elemental, and electrical characterization, they offer limited access to molecular chemical information. AFM-Raman, particularly in its tip-enhanced Raman scattering mode, fills this gap by delivering nanoscale chemical fingerprinting with spatial resolution down to 10–20 nm, enabling simultaneous mapping of molecular composition, crystallinity, strain states, and defect distributions alongside topography. Incorporating AFM-Raman into the correlative metrology toolkit would thus introduce a new chemical information dimension, addressing a longstanding blind spot in existing AFM-SEM integrated systems.

The integration of artificial intelligence (AI) and machine learning (ML) technologies will further assist correlative metrology platforms combining AFM with complementary techniques in achieving automated sample feature identification, image registration, and correlative analysis of multidimensional characterization data [156].

#### 5.4.2. Closing the Loop with Autonomous Process Control

High-speed AFM and ML-based reconstruction have improved throughput, but still rely on manual tuning and offline processing. More critically, ALE’s self-limiting saturation and cryo-etch sidewall quality are validated post-process, not in real time. The long-term goal is an autonomous system where AFM data directly drives etch tool control, enabling self-optimizing processes.

The core of realizing the full AFM-ML feedback loop lies in the deep integration of robust deep learning models driven by synthetic data generation with adaptive AFM control, laying a technical foundation for real-time nanoscale characterization and autonomous discovery workflows [158].

Self-optimizing ALE processes can be advanced by machine learning predictor models, such as transformers, which utilize real-time process data (e.g., pressure) to achieve accurate etch rate prediction, providing a quantitative basis for process regulation [159]. Model training for such etching prediction models requires the adoption of precise dataset aggregation strategies. Utilizing the Pearson Correlation Coefficient (PCC) heuristic to select training data has been shown to enhance model generalization ability in low process variance environments, outperforming other metrics like covariance [159].

The implementation of intelligent characterization and process control systems requires advancing the universal application of synthetic data technology. As demonstrated with SimuScan for AFM, physically faithful synthetic data enables large-scale label-free model training, reducing the development costs of autonomous control systems. Future research should focus on extending such autonomous, synthetic-data-driven workflows to more complex scenarios, including wafer-scale inspection and the characterization of dynamic processes, thereby paving the way for high-throughput, self-optimizing nanomanufacturing [158].

#### 5.4.3. Beyond Silicon: Metrology for Emerging Materials

The evolution of 3D ICs toward true heterogeneous integration—where logic, memory, photonics, and sensors are co-integrated on a single platform—demands metrology solutions that extend well beyond silicon. Advanced nodes increasingly incorporate III-V semiconductors for high-mobility channels and optoelectronics, 2D materials for ultimate thickness scaling, and novel dielectrics for improved isolation and capacitance. Each material class presents distinct etching challenges and requires tailored metrology approaches. Extending advanced AFM metrology to these non-silicon materials has therefore become a pivotal research direction, as precise micro/nanomanufacturing of these materials hinges on reliable etching process monitoring and property characterization.

**Figure 9:**
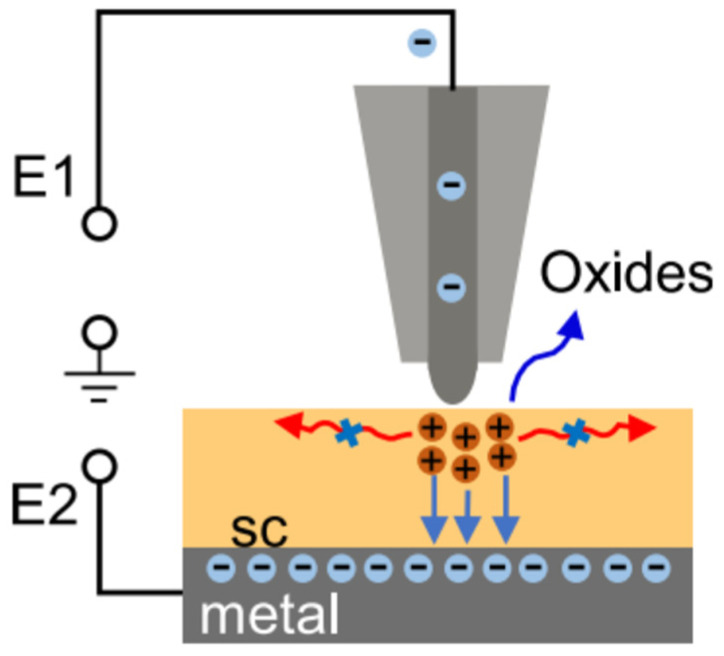
Schematic diagram of the dual-potential machining method, which simultaneously applies an anodic oxidation potential to the probe and a negative bias to the semiconductor substrate in scanning electrochemical probe lithography, achieving high-precision local anodic etching of p-GaAs and other III-V semiconductors. Reproduced from ref. [160] with permission.

For III-V semiconductors: AFM metrology must integrate probe-based potential regulation to address etching-specific challenges. During localized anodic etching of p-GaAs, a dual-potential strategy has been proposed (Figure 9), which suppresses lateral carrier diffusion via a substrate bias, confining the electrochemical reaction to the probe–substrate interface and enabling accurate etching morphology mapping [160].

For 2D materials: probe-based metrology techniques derived from AFM (such as Tip-Enhanced Raman Spectroscopy, TERS) enable nanoscale defect characterization, with demonstrated sensitivity to ion-bombardment-induced defects in MoS_2_ [161]. Parallel probe arrays are being developed to improve throughput for high-volume manufacturing, while maintaining compatibility with CMOS metrology for layer thickness and interface properties post-etching.

For novel dielectric materials (e.g., silicon nitride): AFM metrology can be extended to quasi-atomic layer etching processes, supporting characterization of etching anisotropy and microstructural changes in plasma-modified layers. TEM has revealed high anisotropy, and AFM complements this by providing large-area mapping of anisotropic features. Research in this area should also prioritize the depth and composition metrology of plasma-modified layers, providing data support for the precise regulation of etching ion energy. Furthermore, AFM can be integrated to in situ track the evolution of modified layer thickness during etching cycles [162].

Over the next decade, promoting the lab-to-fab transition of advanced AFM metrology for these materials will require addressing common bottlenecks: for 2D materials, improving AFM throughput via parallel probes; for III-V semiconductors, integrating dual-potential regulation into AFM metrology workflows; and for dielectrics, correlating AFM-derived microstructural data with ion energy tuning.

#### 5.4.4. Bridging Scales: AFM-Informed Multi-Scale Modeling

While AFM provides high-fidelity post-etch topography, multi-scale modeling is indispensable for accessing the dynamic plasma-surface interactions and subsurface phenomena that govern feature evolution yet elude direct experimental observation. However, simulations have long relied on idealized flat surfaces, yet AFM reveals real surfaces are rough, with asperities that affect ion flux, optical scattering, and damage distribution. The next step is to ingest AFM-measured topography into multi-scale frameworks spanning atomic dynamics to feature-scale evolution, enabling predictive process design beyond empirical tuning.

The core development direction of multi-scale modeling integration lies in constructing a seamless simulation workflow that ingests AFM-measured surface topographical data, thereby bridging the hierarchical simulation gap from atomic-scale interfacial interactions (e.g., ion-substrate momentum exchange, chemical bond cleavage) to full feature-scale profile evolution in plasma etching, plasma surface modification, and ultrashort pulse laser ablation [163,164]. This demand stems from the inherent limitation of idealized surface assumptions in conventional simulations, which fail to capture the nanoscale inhomogeneities (e.g., asperities, roughness) that govern real processing outcomes, as highlighted by experimental and simulation discrepancies across all three material processing paradigms.

Capturing atomic-scale interactions relies on MD simulations, which enable atomic-resolution observation of near-surface evolution—including ion-induced collision cascades, chemical etchant adsorption/desorption, and phase transformation dynamics—serving as the foundational atomic-scale module for multi-scale frameworks [164,165]. To bridge atomic-scale insights to feature-scale predictability, coupling MD simulations with reduced order mode (ROM) or continuum mechanics models (e.g., two-temperature model (TTM) and CFD) is imperative: ROM offers orders-of-magnitude faster parameter space exploration by abstracting atomic-scale complexity into mathematically tractable descriptions of transient surface modification [164], while continuum models resolve macroscale phenomena such as lateral heat transport and melt flow that are computationally prohibitive to simulate via MD alone [165].

Seamless integration of AFM data requires quantitative mapping of surface statistical parameters—including RMS roughness, kurtosis, and nanoscale asperity height/distribution—to the initial boundary conditions of multi-scale simulations. This entails abandoning idealized flat-surface approximations to replicate the real surface’s topographical heterogeneity, which directly modulates local energy deposition (e.g., laser fluence scattering, ion flux focusing) and subsequent processing outcomes (e.g., selective ablation, asperity smoothing) [164]. For instance, AFM-derived RMS roughness of 15 nm in aluminum has been shown to induce pronounced optical scattering effects during laser ablation, leading to localized fluence enhancements up to 7-fold and substantially altered crater topographies [165].

The development of such seamless workflows necessitates optimizing the applicability of low-dimensional approximation models (e.g., 1D-TTM) to account for local energy/ion flux inhomogeneities induced by real surface topographies [164,165]. This includes supplementing neglected microscale effects such as lateral electron thermal conduction (triggered by localized fluence gradients) and ion-substrate momentum exchange anisotropy (driven by asperity geometry), which become non-negligible when surface roughness approaches the characteristic length scale of energy deposition (e.g., optical penetration depth, ion collision cascade range) [165].

Over the next decade, establishing a cross-scale parameter calibration system between AFM experimental data and multi-scale model hierarchies is critical. This system should leverage experimentally measured surface profiles, etch/modification efficiencies, and transient response data (e.g., in situ optical emission spectroscopy) to reverse-calibrate atomic-scale interaction potentials (e.g., reactive empirical bond order for MD) and macroscale model coefficients (e.g., electron-phonon coupling constant in TTM). Such a closed-loop calibration will enable improved fidelity of simulations, addressing current limitations such as the discrepancy between MD and ROM predictions in atomic layer etching and the overestimation of surface roughness in laser ablation simulations—thereby paving the way toward truly predictive, material-aware process modeling for advanced semiconductor manufacturing [164,165].

## 6. Conclusions

As 3D IC etching processes continue to diversify—from TSV DRIE to atomic layer etching and cryogenic plasma etching—a critical question emerges: how can metrology keep pace with, and even guide, process innovation? Building upon prior reviews that have offered valuable insights into specific AFM techniques or individual etching processes, this work provides an integrated perspective across the full etching spectrum, revealing the critical bidirectional relationship between metrology capability and process innovation. In this light, AFM emerges not merely as a characterization tool, but as a foundational enabler of advanced etching development, optimization, and industrial adoption—bridging nanoscale topography with functional device performance and serving as a linchpin in correlative metrology and multi-scale modeling. These unique capabilities position AFM as a cornerstone of atomic-scale manufacturing for the next decade of 3D IC scaling. The remaining challenges—throughput, in situ monitoring, and probe durability—are not insurmountable barriers; rather, they represent opportunities to further align AFM’s technical capabilities with the industrial demands of high-volume, atomic-precision semiconductor manufacturing.

## Figures and Tables

**Table 1:** Comparison of AFM with other imaging modalities for semiconductor metrology [22,26,46].

Parameter		Optical Techniques	SEM	TEM	AFM
Resolution	XY	200 nm (conventional) 20 nm (super-resolution) 1–10 nm (near-field optical) <1 nm (Scatterometry, model-dependent sensitivity)	0.5–2 nm	1 nm (conventional) 0.1 nm (aberration-corrected)	1 nm (conventional) 0.1–0.3 nm (UHV non-contact, atomic resolution) <0.5 nm (PeakForce Tapping, optimized conditions)
	Z	about 1 nm (technique-dependent)	Tilt-Beam: sidewall angle precision ~0.1–0.2° (for 290 nm height features) Model-Based: Z information derived from library fits	NA	0.1 nm (typical) 0.00037 nm (ultra-high sensitivity)
Sample Preparation		Simple	Moderate	Skilled (electron transparent required)	Simple
Ambient Atmosphere		Variable (air, vacuum)	Vacuum	Ultra-high Vacuum	Variable (air, liquid, vacuum)
Field of View		Large (10 μm to mm scale)	Large enough (50 nm to 10 mm)	Limited (tens of nm to ~200 μm)	Moderate (tens of nm to >500 μm)
Functionality		Moderate to Advanced (interferometry, scatterometry, confocal, structured light)	Moderate (imaging, EDS, EBSD)	Moderate (imaging, diffraction, spectroscopy)	Advanced (mechanical, electrical, magnetic, thermal)
Speed		Fast (in-line, wafer-level mapping)	Moderate (move-acquire-measure time < 6 s per site)	Slow (FIB lamella prep ~20 min + imaging ~25 min) Fast (automated CD-TEM: <10 s per CD measurement)	Slow (conventional) Fast (high-speed AFM: video-rate, 10–20 fps; CD-AFM: throughput limits large sampling)
Skill Required		Low	Moderate	Advanced	Advanced
Data Interpretation		Moderate to complex (model-dependent)	Moderate	Moderate	Complex (convolution, artifacts)
3D Profile Capability		Limited (line-of-sight)	Limited	Cross-section only	Yes
Non-destructive		Yes	Yes (potential damage at high voltage)	No (requires destructive sample preparation)	Yes
Sidewall Access		Limited	No	Cross-section only	Yes

## Abbreviations

The following abbreviations are used in this manuscript:

3D	three-dimensional
3D-AFM	three-dimensional atomic force microscopy
3D-SICs	3D stacked ICs
AAO	anodic aluminum oxide
AFM	atomic force microscopy
AFP	Atomic Force Profiler
AI	artificial intelligence
ALD	atomic layer deposition
ALE	atomic layer etching
APT	atom probe tomography
BEOL	backend-of-line
CCP	capacitively coupled plasmas
CD	critical dimension
CD-SAXS	critical dimension small-angle X-ray scattering
cryo-ALE	cryogenic atomic layer etching
CMOS	Complementary metal-oxide-semiconductor
CMP	chemical mechanical polishing
CNM	compliant nano-manipulator
CPEM	Correlative Probe and Electron Microscopy
CW	continuous wave
DRIE	Deep Reactive Ion Etching
eCD	enhanced CD
EELS	electron energy loss spectroscopy
EFEM	equipment front end module
ETD	estimated tip diameter
F2F	face-to-face
FA	fast scanning actuator
FC	fluorocarbon
FFTP	Feed Forward Trajectory Planning
FIB	Focus Ion Beam
FinFETs	Fin Field-Effect Transistor
GAA	gate-all-around
HAR	high-aspect-ratio
HARPs	High-Aspect-Ratio Probes
HBM	high-bandwidth memory
HHCF	height-height correlation function
HS-AFM	high-speed AFM
HS Met. LR-AFM	high-speed metrology large-range AFM
ICP	Inductively Coupled Plasma
ICs	integrated circuits
IE-AM	Image Explorer for Advanced Metrology
LER	Line Edge Roughness
LWR	line width roughness
M3D	monolithic 3D integration
MAFM	miniaturized AFM
MEMS	microelectromechanical System
ML	machine learning
MOS	metal-oxide-semiconductor
MOSFET	Metal-Oxide-Semiconductor Field-Effect Transistor
MWCNT	multiwall carbon nanotube
NBE	neutral beam etching
OCD	optical critical dimension
OES	Optical Emission Spectroscopy
OFP	optical fiber probes
OPC	optical proximity correction
PECVD	Plasma-Enhanced Chemical Vapor Deposition
PSD	Power Spectral Density
QALE	quasi-atomic layer etching
$R_{a}$	average roughness
$R_{q}$	root mean square roughness
RMS	root mean square
ROM	reduced order mode
$R_{p-p}$	peak-to-peak roughness
$R_{sk}$	skewness
$R_{ku}$	kurtosis
SAMs	self-assembled monolayers
SCEs	short-channel effects
SEM	scanning electron microscopy
SiP	system-in-package
SNOM	scanning near-field optical microscope
SoC	system-on-chip
SPMs	scanning probe microscopes
SWA	sidewall angle
TEM	transmission electron microscopy
TERS	Tip-Enhanced Raman Spectroscopy
tilting-mAFM	metrological tilting-AFM
TRS	transitional rescan
TSVs	through-silicon vias
TTM	two-temperature model
ULK	ultra-low-κ
ULSS-AFM	Ultra-large scan size AFM
VIA	Vertical Interconnect Access
VPS	vector probing scanning
WIW	within-wafer
